# Uptake of DU145 and LNCaP prostate cancer cell line derived extracellular vesicles is inversely correlated with blood–brain barrier integrity in vitro

**DOI:** 10.1186/s12987-025-00680-7

**Published:** 2025-07-07

**Authors:** Ana Špilak, Adrián Klepe, Sophia Theresa Kriwanek, Heinz-Peter Friedl, Andreas Brachner, Christa Nöhammer, Winfried Neuhaus

**Affiliations:** 1 Competence Unit Molecular Diagnostics, Center for Health and Bioresources, AIT-Austrian Institute of Technology GmbH, Giefinggasse 4, 1210 Vienna, Austria; 2 Faculty of Medicine and Dentistry, Danube Private University, 3500 Krems, Austria

**Keywords:** Small extracellular vesicles, Prostate cancer, Biological barriers, Blood–brain barrier, Inflammatory cytokines

## Abstract

**Background:**

Tumor-derived small extracellular vesicles (sEVs) have been implicated in changes of the blood–brain barrier (BBB) during pre-metastatic niche formation. Although it was postulated that sEVs can traverse the highly restrictive BBB via transcytosis—data mostly based on the indirect detection of transported cargo—direct evidence for sEV transport across the BBB remains elusive due to challenges in sEV labelling, detection limits, and inherent limitations of existing in vitro BBB models. This study investigated the interaction and effects of sEVs derived from low (LNCaP) and moderately metastatic (DU145) prostate cancer (PCa) cell lines with the human brain endothelial cell line hCMEC/D3.

**Methods:**

Systematic optimization of the cell culture membrane insert set-up for sEV transport studies was accomplished with inserts with different pore sizes, varied coating procedures and medium compositions. Particle size distribution, quantification and zeta-potential was measured with nanoparticle tracking analysis. Uptake of fluorescent labelled sEVs by hCMEC/D3 cell layers was determined by flow cytometry, barrier integrity was measured by transendothelial electrical resistance (TEER). Effects of inflammatory cytokines and PCa lines-derived sEVs on hCMEC/D3 at the transcriptomic level were investigated by means of high-throughput qPCR based on Fluidigm Biomark® platform.

**Results:**

Improved conditions for sEV transport studies included the application of membrane inserts with 1 µm pore size and of 1% BSA in the receiver compartment. Efficiency of LNCaP- and DU145-derived sEV uptake by hCMEC/D3 cells revealed an inverse correlation between uptake of sEVs and paracellular barrier integrity (TEER). Whereas addition of sEVs of the more aggressive DU145 cells resulted in a distinct increase of TEER under regular and inflammatory conditions, LNCaP-derived sEVs affected TEER only upon inflammatory cytokine treatment. MRNA expression analyses of hCMEC/D3 cells revealed a distinct regulation of transcripts depending on TEER (i.a. FN, CDLN1) or upon inflammatory cytokines (i.a.: ABCB1, MFSD2a, VCAM1, VEGFa).

**Conclusions:**

Differences upon treatment of hCMEC/D3 layers with LNCaP-and DU145 derived sEVs indicated that vesicles retain and transport molecular features of their originating cells. Careful optimization of the test set-up for studies with sEVs in vitro is recommended, including medium controls for sEV purification and labelling as well as addition of proteins for sEV recovery.

**Supplementary Information:**

The online version contains supplementary material available at 10.1186/s12987-025-00680-7.

## Background

Small extracellular vesicles (sEVs), including vesicles formerly defined as exosomes, are secreted by virtually all cell types, and are characterised by their size (diameters between ~ 50 and 200 nm) and their biogenesis via multivesicular bodies. They are present in body fluids such as blood, urine, and saliva, both under physiological and pathological conditions [91, 101, 102]. Initially considered to be a cellular waste-disposal mechanism [14], a plethora of extracellular vesicle studies revealed their role in intercellular communication by transferring bioactive components (nucleic acids, proteins, and lipids) from donor to recipient cells [5, 22, 25, 47, 90]. A limited number of reports suggested that cancer-derived sEVs interact with endothelia [8, 48] and promote cancer progression by formation of pre-metastatic niches [66, 85], but detailed molecular mechanisms and causal effects induced by the interaction of cancer-derived sEV with endothelial barriers have not been elucidated so far.

Prostate cancer (PCa) stands out as the most diagnosed cancer in men, being responsible for 10% of all cancer-related deaths in the recent years [74]. Beyond its most frequent target organs, i.e. bones and lymph nodes, in rare cases (< 2%) PCa metastasizes also to the brain, capable invading through the blood–brain barrier (BBB) [29, 87]. A first clinical trial demonstrated the potential of sEVs in urine samples as biomarkers to detect such high-grade (invasive) prostate cancers [89], and extensive research has deciphered the molecular composition of PCa sEVs [45]. Their putative role in facilitating the spread of cancer cells across biological barriers, including the BBB, has not been studied yet [54, 85].

The BBB protects the central nervous system (CNS) from potentially noxious agents present in the bloodstream, facilitates, and regulates the exchange of molecules between peripheral blood and CNS, and is crucial for the maintenance of CNS homeostasis. The BBB is formed by a continuous, non-fenestrated monolayer of brain capillary endothelial cells (BCECs), which are interconnected by tight- and adherens junctions [50, 98]. The polarized BCECs interact on the basolateral (brain) side with astrocytes, pericytes and neurons, forming the so-called neurovascular unit (NVU), an intricate regulatory network modulating BBB functions [52]. Due to this tight paracellular barrier and the low rate of transcytosis (endocytosis, intercellular trafficking, and exocytosis) as compared to peripheral vascular endothelia [2], the transport of most molecules across the BBB is regulated via active mechanisms, including ABC and SLC transporters and receptor or adsorption mediated processes. Similar restrictions might apply to uptake and transport of sEVs at the BBB [61].

In cancer pathology, inflammation is an important and well-known factor in all stages of tumor development, driving tumor initiation, promotion, and progression. About 20% of adult cancers can be attributed to inflammatory diseases [49], chronic prostate inflammation doubles the risk of developing PCa later in life as compared to healthy men [73]. Pro-inflammatory cytokines (such as IL-1β, IL-6, TNFα, and IFN-γ) secreted during systemic inflammation upon cancer progression, were shown to compromise BBB properties both in vitro and in vivo by increasing BBB permeability and induced further or worsened pathological conditions [7, 26, 60, 92].

Several studies have addressed uptake and transport of sEVs at the BBB, testing human primary brain microvascular endothelial cells (BMEC) with HEK293 derived sEVs [12], mouse in vivo and human in vitro BMEC models with red blood cell- [51], astrocyte- [16], or breast cancer-sEVs [54], mouse in vivo and monkey in vitro BMECs with breast cancer-sEVs [85], and porcine primary BMECs and hCMEC/D3 cells with sEVs isolated from human plasma [33]. Further relevant studies investigated uptake and/or transport in hCMEC/D3 cells with neutrophil-derived EVs [1], cytokine-treated hCMEC/D3-sEVs [67], melanoma-derived sEVs [38], neural stem cell sEVs [34] and bacterial EVs [53].

Overall, these studies showed that EVs from very different origins (and their respective cargoes) are up taken by primary BMECs and the brain endothelial cell line hCMEC/D3. In addition, results from in vivo and few in vitro models suggested that EVs (or their cargoes) can cross the BBB. Whereas the mechanism of sEV uptake was narrowed down experimentally to endocytic pathways already [12, 21], the pathway of vesicle transport across the BBB has not been elucidated yet.

Preliminary experiments have shown that cell culture membrane inserts can pose a significant physical obstacle for sEV permeation, hence confounding results obtained from such model setups. In addition, sEVs, as many other lipophilic compounds, may adsorb to plastic surfaces, leading to a diminished number of bio-available vesicles in in vitro cell culture systems. In order to perform PCa-sEV BBB studies under optimal experimental conditions, an extensive evaluation of the employed membrane insert-based model was conducted, including the comparison of different membrane pore sizes, surface coatings and media supplements. With the such improved hCMEC/D3-based BBB model, the uptake, permeation, and effects of sEVs derived from two human PCa cell lines, LNCaP [31] and DU145 [79] on barrier integrity and recipient cells’ gene expression were assessed, including setups with inflammatory cytokines.

Surprisingly, treatment of hCMEC/D3 cells with sEVs from the more aggressive cell line DU145 led to higher barrier integrity, while LNCaP sEVs had no effect as compared to controls under normal conditions, but resulted in higher barrier tightness as well, when inflammatory cytokines were present in the medium. Secondly, efficiency of sEV uptake and barrier integrity showed an inverse correlation, suggesting an interplay between cellular uptake mechanisms and pathways establishing paracellular barrier tightness. Finally, despite that cellular uptake of sEVs was readily detected, the number of EV-sized particles found to have crossed the endothelial cell layer was not reliably detectable by single particle tracking analysis.

## Methods

### Cloning and preparation of plasmids

The DasherGFP-glycosyl-phosphatidyl-inositol (GPI) expression plasmid (GFP-GPI) was generated by ligating a synthetic DNA fusion construct encoding in frame a signal peptide (SP) sequence, the DasherGFP open reading frame (#FPB-27-609; ATUM, Newark, CA, USA), V5 tag (V5) and the GPI motif of CD55 (Decay-accelerating factor) into the pUC19 vector. The SP-DasherGFP-V5-GPI fusion construct was excised from pUC19 with BsaI and ligated into the BsaI sites of the pCMV mammalian expression vector (ATUM, Newark, CA, USA). The construct was verified by Sanger sequencing. Plasmid DNA for cell transfections was extracted from transformed E. coli using the FavorPrepTM Endotoxin Free Midi kit (#FAPDE 002-EF; Favorgen Biotech Corp, Ping Tung, Taiwan).

### Cultivation and transfection of HEK293 cells

HEK293 cells were cultured in Dulbecco’s Modified Eagle Medium (DMEM; #D5796; Sigma-Aldrich St. Louis, MO, USA) supplemented with 10% foetal bovine serum (FBS; #F9965; Sigma-Aldrich) and 100U/mL Penicillin, 100 µg/mL Streptomycin (P/S; #A2213; Merck, Darmstadt, Germany) at 37 °C in a humidified atmosphere containing 5% CO_2_. Cells were routinely split at a confluency of 80–90% using 0.25% trypsin/EDTA (#L2143; Merck) for cell detachment. The HEK293-GFP-GPI cell clone was established by polyethyleneimine (PEI; 23966-1, Polysciences)-mediated transfection of the pCMV-SP-Dasher-V5-GPI plasmid into HEK293 cells. The transfection was conducted in a 100 mm petri dish (PD10; #664160; Greiner Bio-One, Kremsmünster, Austria) by incubation of a 70% confluent culture of HEK293 cells with a mix of 12 µg plasmid DNA and 36µL PEI in 1.2 mL OptiMEM™ (#10149832; Gibco™—Thermo Fisher Scientific, Waltham, MA, USA) for 24 h. Thereafter, stably transfected cells were enriched by addition of 1 µg/mL puromycin to the growth medium for 1 week and subsequently GFP-positive clones were selected using cloning cylinders (#YA79.1; Carl Roth, Karlsruhe, Germany) and expanded. Expression and localization of the recombinant protein was confirmed by fluorescence microscope, flow cytometry and Western blotting (Figure S1).

### Cultivation of DU145 and LNCaP cells

PCa cell lines were kindly provided by the Microenvironment and Metastasis Group, Spanish National Cancer Research Centre (Madrid). DU145 cells were maintained in DMEM supplemented with 10% FBS, 1% sodium pyruvate (#S8636; Merck) and P/S. Cells were trypsinized and split (1:6–1:8) two times per week. LNCaP cells were cultivated in RPMI-1640 (#R8758; Sigma-Aldrich) containing 10% FBS and P/S and passaged weekly (1:3–1:6).

### Cultivation of hCMEC/D3 cells

The human immortalised cell line hCMEC/D3 [95] was obtained from Merck Millipore (#SCC066) and cultured as described previously [23]. In brief, hCMEC/D3 were grown on 0.5% gelatine-coated culture flasks (#22.151.02,SERVA Electrophoresis GmbH, Heidelberg, Germany in EBM-2 endothelial basal media (#CC3156; Lonza, Basel, Switzerland supplemented with 5% FBS, P/S, 10 mM HEPES (#H0887; Sigma-Aldrich, 5 µg/mL ascorbic acid (#A4544; Sigma-Aldrich; stock solution: 1 mg/mL ascorbic acid in EBM-2 medium and 1 ng/mL human-basic fibroblast growth factor (hbFGF; #F0291; Sigma-Aldrich; stock solution: 200 ng/mL hbFGF in DPBS containing 0.1% BSA. hCMEC/D3 cell cultures were split weekly at full confluence at a ratio of 1:3.

### Small EV purification

HEK293-GFP-GPI, DU145 and LNCaP cell lines were cultivated in 145 mm cell culture dishes (PD145; #639160; Greiner Bio-One). When the cells reached 60–70% confluency, cells were washed once with DPBS (#14190-144; Thermo Fisher Scientific) and further incubated in 25 mL sEV collection medium for 24 (HEK293-GFP-GPI) or 48 h (PCa cells). Collection media for HEK293 cells was composed of Pro293a, chemically defined, serum-free medium (#BEBP12-764Q; Lonza) supplemented with 2 mM l-Glutamine and P/S, serum-free maintenance media served for collection from PCa cells (Figure S2).

Cell culture supernatants were collected and floating cells, cell debris, apoptotic bodies, and larger vesicles were depleted by differential centrifugation at 4 °C: (1) 10 min at 300×*g*, (2) 15 min at 2000×*g*, (3) 25 min at 10,000×*g*. Cleared supernatants were concentrated using Amicon ultrafiltration units with 100 kDa (HEK293-GFP-GPI) and/or 10 kDa (DU145 and LNCaP) cutoff (#UFC810096, #UFC801096; Sigma-Aldrich). Ultrafiltration units were centrifuged at 4000×*g* up to 1 mL of enriched sEV medium retentate volume was reached. Small EVs were then purified by size exclusion chromatography (SEC) using EX04 Exo-Spin midi columns (#EX04-20; Maxanim, Gentaur, Aachen, Germany) according to the manufacturer’s instructions. Fractions 7–12 were collected in DPBS in Protein LoBind® Tubes (#022431081, Eppendorf, Hamburg, Germany). The isolated sEVs suspensions were concentrated using 100 kDa (HEK293 GFP-GPI) and/or 10 kDa (DU145 and LNCaP) Amicon-Ultra 0.5 centrifugal filter tubes (#UFC510096, #UFC501096; Sigma-Aldrich) by centrifugation at 10,000×*g* until a final volume of 100–200 µL retentate volume was reached, which was collected by inverting the tube and centrifuging at 1000×*g* for 2 min. Before freezing and storing sEVs at − 80 °C, size, number, and zeta potential of sEVs were determined by Nanoparticle Tracking Analysis (NTA) using a ZetaView® Quatt device (Particle Metrix GmbH, Inning am Ammersee, Germany). Serum-free media were incubated in petri dishes without cells and underwent the exact same purification process as cell-derived sEVs and served as negative controls (“media controls”) in all experiments (Figure S3).

### Fluorescent labelling of PCa sEVs

A 10 mM stock solution of CellTracker™ Orange dye (CTO, #C34551; Invitrogen) in DMSO was prepared and diluted in DPBS to achieve a 20 µM working solution, which was mixed 1 + 1 part with the sEV suspension (10 µM CTO final concentration) and incubated light-protected, with gentle shaking, for 3 h at 37 °C. Residual free dye was removed by adding twice a 20-fold volume of DPBS after labelling and purification via ultrafiltration with 100 kDa Amicon-Ultra 0.5 centrifugal filter tubes as described before. Particle concentration, size distribution and labelling efficiency was determined by NTA. The same procedure was done in parallel with media controls.

### Nanoparticle tracking analysis

The hydrodynamic diameter (“size”), concentration, zeta potential and fluorescent label of sEVs were measured and analysed using a ZetaView® Quatt device and the software ZetaView® 8.05.12 SP2. All samples were diluted in ultrapure water to achieve a concentration of 50–200 particles per field of view, ensuring reliable measurements as recommended by the manufacturer. For each scatter measurement (size or zeta potential), one to three cycles, while in fluorescence mode (F-NTA), one cycle, at 11 positions each and capturing 30 (scatter mode) or 15 frames (fluorescence mode) per second were recorded. In scatter mode, the measurement parameters “sensitivity” and “shutter” were set to 75 and 100, respectively. The analysis parameters were defined as: Maximum particle size = 1000, minimum particle size = 10, and minimum particle brightness = 30. Fluorescence measurements of GFP-positive sEVs were performed with following settings: Sensitivity = 92 and shutter = 75, with the “low bleaching” option of the device activated. CellTracker® Orange labelled samples were measured at sensitivity = 92 and shutter = 95. The minimum particle brightness in fluorescence mode was set to 25. Values are shown as mean number of particles. Due to high background of media supplemented with BSA, the shutter had to be raised to 350 in scatter mode, and 100 in fluorescence mode. All measurements were done at 24 °C set in the instruments SOP.

### Calculation of sEV permeation and recovery

Actual sEV numbers related to the volumes of the respective donor and receiver compartments in the transwell models were calculated for scatter and fluorescent measurement results. Permeation of sEVs was calculated by dividing the number of particles in the receiver compartment by the sum of particles in the donor and receiver compartment (shown as percent of the total particle number which permeated in Fig. 2, Figs. S4, S6). For the recovery calculation, the sum of the actual sEV numbers both from the donor and receiver compartments were divided by the volume-adjusted, applied sEV number.

### Cryo-transmission electron microscopy

4µL of highly concentrated sEV DPBS suspensions (1.85 × 10^11^, 3.3 × 10^11^ and 2.4 × 10^11^ sEVs/mL of HEK293-GFP-GPI, DU145 and LNCaP, respectively) were loaded on copper grids with 2 µm pores and snap-frozen in liquid ethane at − 188 °C using a fully automated plunge freezer. sEV cryo-EM imaging was performed by on Glacios™ Cryo-TEM (Thermo Fisher Scientific) at 150,000× magnification (pixel size: 0.9719 Å). All sample preparation works, and imaging were performed by the Electron Microscopy Facility at Vienna BioCenter Core Facilities (VBCF), member of the Vienna BioCenter (VBC), Austria.

### Western blotting

Western blotting was conducted with slight modifications as published previously [55]. In brief, after harvesting media for sEV purification, cells were washed twice with 5 mL of ice-cold DPBS and then incubated in 900 µL RIPA buffer (50 mM Tris–HCl pH 7.4 (#5429.3; Carl Roth), 150 mM NaCl (#0241; VWR International, Vienna, Austria), 150 mM EDTA (#E1644; Sigma), 1 mM Triton™ X-100 (#T8787, Sigma-Aldrich), 0.01% SDS (10% SDS solution, #A0676,1000; PanReac AppliChem, Darmstadt, Germany) supplemented with protease inhibitor cocktail (Roche cOmplete ULTRA tablets mini, #05892970001; Sigma-Aldrich) and phosphatase inhibitors (Roche PhosphoSTOP, #04906837001; Sigma-Aldrich) per PD145 dish, on ice. After 20–25 min cells were scraped, and lysates were collected, sonicated on ice with a tip sonicator (Fisherbrand™ Q505 sonicator; Thermo Fisher Scientific; settings: 30% amplitude, 3 pulses, 1 s per pulse), and stored at − 80 °C until further use. Protein concentrations were determined either using the Pierce BCA or micro-BCA Protein Assay kit (#23225, #23235; Thermo Fisher Scientific) depending on expected protein concentrations.

Prior to sample loading, Laemmli buffer was added to the lysates, and proteins were denatured by boiling for 5 min at 95 °C. 20 µg of total protein was loaded per lane of 10% SDS–polyacrylamide gels, and gel electrophoresis was performed at 130 V. Proteins were transferred over night at 4 °C onto polyvinylidene difluoride membranes (#162-0177; Bio-Rad Laboratories, Hercules, CA, USA) using a tank blotter (Mini Protean; Bio-Rad) at 40 mA constant current per each membrane. Membrane blocking, incubations with primary and secondary antibodies and signal detection were carried out as described previously [55]. Applied antibodies are listed in Table S1.

### Cell culture membrane insert experimental setups

All experiments in this study were performed with Greiner ThinCert® membrane inserts in 24-well plate formats, for setup optimization, membranes with 0.4, 1.0 and 3.0 µm pores were included (#662641, #662610, #662630; Greiner Bio-One). ThinCerts® were coated by incubating the membranes apically with 100 µL of a mixture of 0.1 mg/mL collagen IV and 1 mg/mL fibronectin in DPBS (#C5533, #F1141; Sigma-Aldrich; the 1 mg/mL collagen IV stock solution was prepared by dissolving lyophilized human collagen IV in 0.005% acetic acid at 4 °C overnight), either for 4 h or overnight at 37 °C. After incubation with the coating mixes, inserts were washed twice with sterile DPBS and immediately used for experiments.

Inserts were incubated with serum-free, P/S containing EBM-2 medium, or additionally supplemented with 5% FBS, (300 µL apically, 900 µL basolaterally) for 24 h, followed by a medium change to EBM-2 containing 0%, 0.25%, 0.5% or 1% BSA, and P/S. In blank insert studies, sEVs were added apically, and apical and basolateral media were collected after 24 h incubation at 37 °C. Permeation and recovery of sEVs were calculated based on NTA measurements. Each experiment was repeated twice or thrice, with two technical replicates of each condition in experiments using 0.4 and 1.0 µm inserts and 1–3 when 3.0 µm inserts were tested.

Transwell insert experiments with hCMEC/D3 cells were carried out as described before [23, 77] with the optimised experimental system for sEV studies. Briefly, Greiner 24-well ThinCerts® with a pore size of 1.0 µm were coated with a collagen IV-fibronectin mixture as described above. hCMEC/D3 were seeded at a density of 40,000 cells/cm^2^. Medium was changed on day 3 and 6; on day 6 serum in the medium was reduced to 0.25% FBS. For experiments, 5 × 10^9^ CTO-labelled sEVs per mL or the equivalent volume of medium control was added apically to the EBM-2 medium. On the basolateral side serum-free EBM-2 or medium supplemented with 1% BSA or 0.25% FBS was used. Inflammatory conditions were induced by adding a mixture (named CYTO) of each, 100 ng/mL, tumor necrosis factor alpha (TNFα, #H8916; Sigma-Aldrich; stock solution: 10 µg/mL in DPBS containing 0.1% BSA), interferon gamma (IFN-γ, #300-02; PeproTech, Thermo Fisher Scientific; stock solution: 10 µg/mL in DPBS containing 0.1% BSA) and interleukin 1-beta (IL-1β; 200-01B-C3122, PeproTech, Thermo Fisher Scientific; stock solution: 10 µg/mL in DPBS containing 0.1% BSA), together with sEVs, apically to the cells. Each experiment was repeated three times, with three replicates for each condition per experiment.

### Transendothelial electrical resistance measurement

Transendothelial electrical resistance (TEER) measurements were performed with an EVOM-3 voltohmmeter device (World Precision Instruments, Sarasota, FL, USA) and STX2 chopstick electrodes (Merck Millipore). Measurements were done after media exchanges and a temperature equilibration time of 40 min at room temperature. The initial TEER (timepoint 0 h) was determined after addition of the experimental media and temperature equilibration. TEER values, given as Ω × cm^2^, were calculated by subtraction of the mean resistance value (Ω) of three blank ThinCerts® and multiplication with the growth surface area (0.336 cm^2^).

### Flow cytometry

Flow cytometry of hCMEC/D3 cells was performed on a CytoFLEX device (Beckman Coulter, Brea, CA, USA) calibrated routinely with CytoFLEX daily QC Fluorospheres (#B53230; Beckman Coulter) according to the manufacturer’s instructions. Live singlet cells were gated using the forward and side scatter signals, and CTO positive cells were determined using the PE channel settings of the instrument (excitation wavelength 488 nm; emission detection using the bandpass filter 585/42BP). hCMEC/D3 cells stained with 10 µM CTO dye for 15 min at room temperature and cells treated with media controls served as positive and negative controls, respectively. Flow cytometric data were processed with the Kaluza Analysis software 2.1 (Beckman Coulter). The average median fluorescence intensities (MFI) of untreated controls were subtracted as background from the MFI values of media controls and sEV samples, yielding background-corrected values used for normalization and further calculations.

### Quantitative real-time PCR and high-throughput qPCR

Total RNA was isolated from cells lysed in RLT buffer containing 1% β-mercaptoethanol using the “All prep DNA/RNA/protein mini kit” according to the manufacturer’s instructions (#80004; Qiagen). 900 ng of total RNA was reverse transcribed to cDNA using the Applied Biosystems™ “High-capacity reverse transcriptase kit” (#4374967; Thermo Fisher Scientific) according to the manufacturer’s instructions. qPCR amplification of Vascular Cell Adhesion Molecule 1 (VCAM1 transcript variant a), Intercellular Cell Adhesion Molecule 1 (ICAM1, transcript variant b), nuclear factor κB (NF-κB) RELA, TNF Receptor Associated Factor 2 (TRAF2, transcript variants b and c), Janus Kinase 1 (JAK1, transcript variants a, b, and c) were performed on a LightCycler480 II device (Roche) as described before [43]. The housekeeping genes 18S rRNA and β-actin were amplified for normalization. Primer sequences are listed in Table S2. Each sample was analysed as technical triplicate. Relative gene expression levels were calculated using the 2^−∆Cq^ method.

High-throughput qPCR was performed with cDNA reverse-transcribed from 250 ng of total RNA and preamplification of target genes using a Qiagen PCR master mix with HotStar Plus Taq Polymerase (#203603, Qiagen) as described before [23, 62, 77]. The high-throughput qPCR was run with the preamplified cDNAs in a 96 × 96 microfluidic chip setup on a Biomark HD™ system (Fluidigm®). The investigated 88 target- and 4 housekeeping genes are listed in Table S3. Threshold cycle (Cq) values were normalised to the geometric mean of the Cq values of the housekeeping genes (PPIA, ACTB, GAPDH and B2M). Relative gene expression levels were calculated and visualised in a heatmap displaying the log2-fold changes normalised to the respective sEV-treated samples using the Qlucore Omics Explorer 3.8 software (Qlucore, New York, USA). The value of each target was scaled to the range of − 2 to + 2 for visualisation.

### Statistics

Results are shown as mean ± SEM unless stated otherwise. Statistical tests (Student’s t-test or one-way ANOVA followed by pairwise Holm-Šidak’s multiple comparison post hoc tests) and graphical visualisations were done with GraphPad Prism 8.0 (Dotmatics, Boston, MA, USA). Statistical outliers were identified using the Grubb’s test and removed from final calculations. For all statistical comparisons, an alpha value of 0.05 was used. Differences were considered statistically significant at p < 0.05. High-throughput qPCR results were analysed using 2^−∆Cq^ values. In order to compare multiple genes between two test groups, means were compared by Kruskal–Wallis’s tests followed by Dunn’s multiple comparison post hoc test.

## Results

### Characterisation of sEVs

**Fig. 1:**
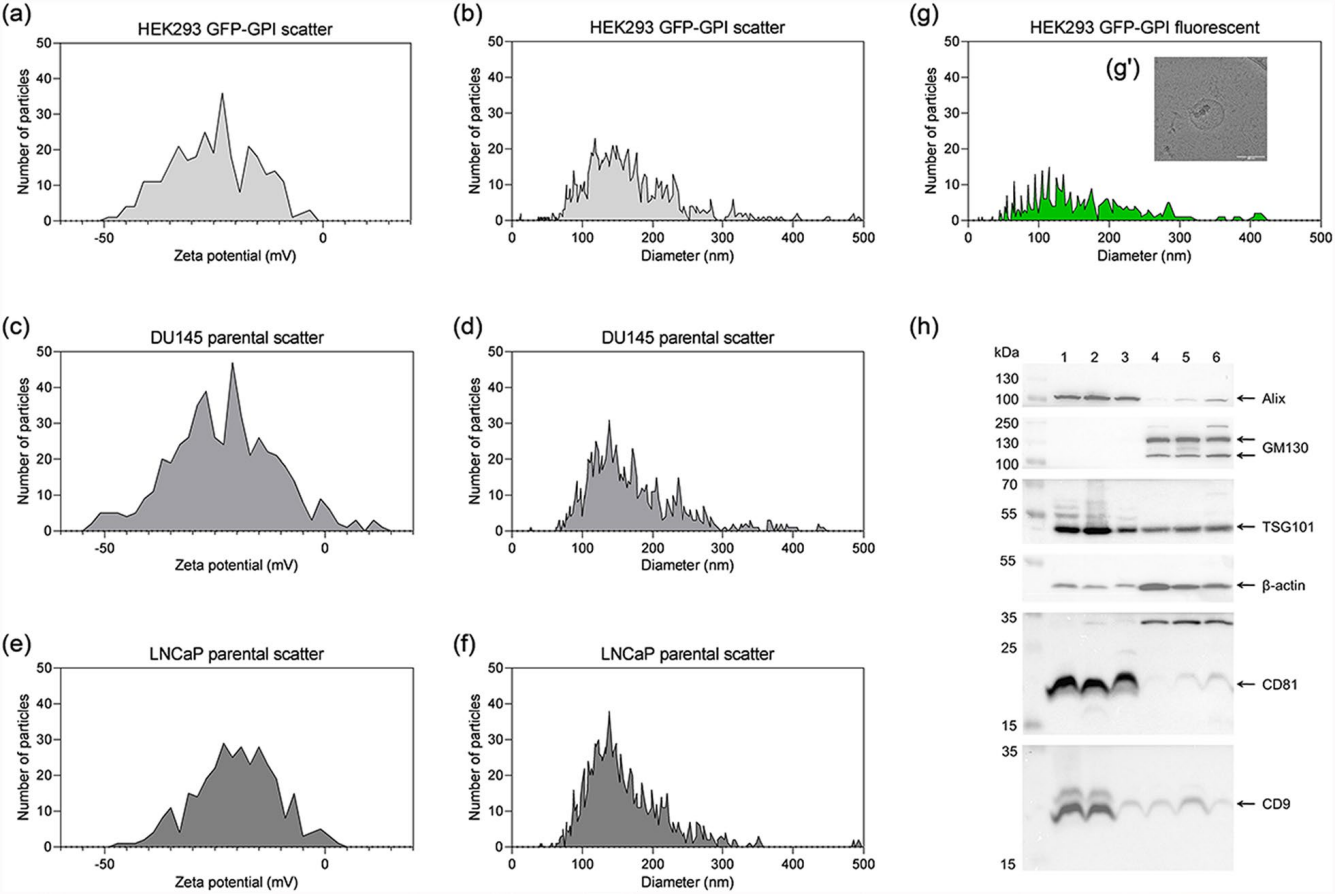
Characterisation of sEVs derived from **a**, **b**, **g** HEK293 GFP-GPI transfected, **c**, **d** DU145 and **e**, **f** LNCaP parental cells. Representative graphs of **a**, **c**, **e** sEV zeta potential their size distribution (**b**, **d**, **f**) in scatter mode and **g** in fluorescent mode using (F-)NTA. **g'** Representative image of a single HEK293 GFP-GPI double membrane bound vesicle observed by cryo-TEM. Scale bar = 100 nm. **h** Expression of sEV markers, CD9, CD81, TSG101 and Alix and negative marker GM130 and β-actin confirmed by Western blotting. On each lane 20 µg of protein sample was loaded as follows: 1—DU145 sEVs, 2—LNCaP sEVs, 3—HEK293 GFP-GPI sEVs, 4—DU145 cell lysate, 5—LNCaP cell lysate, 6—HEK293 GFP-GPI cell lysate. On the left side of the membrane blots, the ladder in kDa is labelled and on the right side, the expressed markers are added

Small EVs were purified from HEK293 cells stably expressing a GFP-GPI construct (Figure S1), and from two PCa cell lines (DU145 and LNCaP), using a combination of ultrafiltration and size exclusion chromatography (Figure S2). Following the MISEV guidelines for EV characterization [96], the hydrodynamic diameter (size) and surface charge (zeta potential) of the purified vesicles were determined by nanoparticle tracking analysis (NTA), revealing median sizes and surface charges as typically reported for sEVs: 138 ± 2.7 nm, − 21.1 ± 1.8 mV (HEK293-GFP-GPI), 145 ± 0.9 nm, − 21.6 ± 1.6 mV (DU145) and 137 ± 0.7 nm, − 20.5 ± 0.9 mV (LNCaP) (Fig. 1, Table S4).

Furthermore, the expression of sEV marker proteins, including tetraspanins (CD9, CD81) and cytosolic proteins (TSG101, Alix) were confirmed by Western blots, while the Golgi protein GM130 served as a negative marker (Fig. 1h). The vesicular nature of the isolated samples was visualized by cryo-TEM, clearly revealing the double lipid membrane in a representative HEK293-GFP-GPI sEV sample (Fig. 1g’). Taken together, the established methodology yielded pure extracellular vesicles with characteristics defining sEVs [83, 96].

### Optimisation of the transwell insert model for sEV permeation studies

Previous studies have addressed sEV permeation and sEV-cell interaction with BBB cells in various cell culture membrane setups [63], but systematic testing of technical parameters affecting vesicle permeation across such in vitro models was not reported yet.

**Fig. 2:**
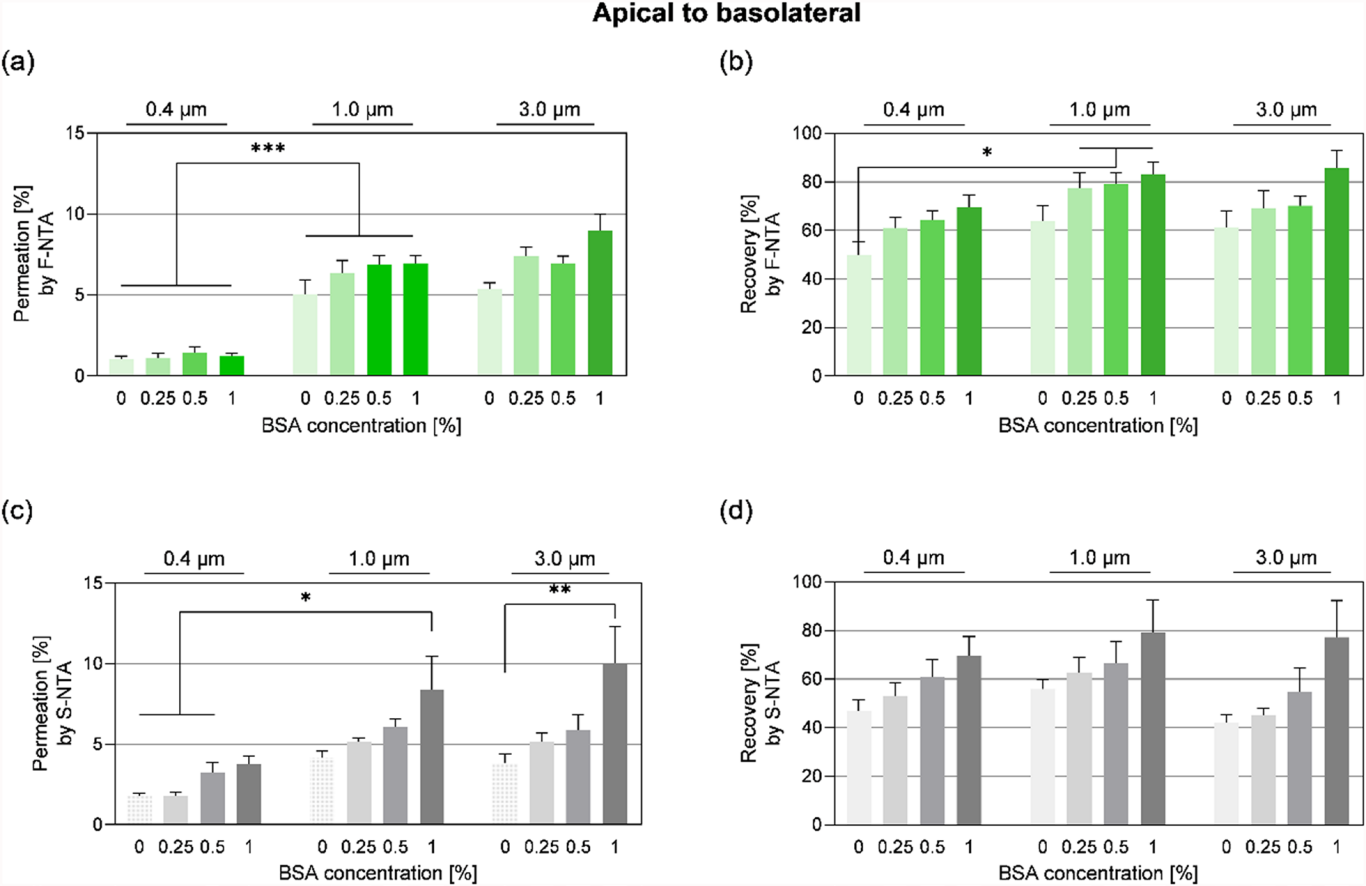
Addition of Bovine Serum Albumin (BSA) increases detectable number of sEVs using 10^9^ sEVs per mL derived from HEK293 GFP-GPI in transport studies across blank 0.4 µm, 1.0 µm and 3.0 µm pore size inserts in EBM-2 medium supplemented with 100 U/mL P/S in a concentration dependent manner after 24 h. **a**, **c** Effect of presence of 0%, 0.25%, 0.5% and 1.0% BSA on sEV diffusion from apical to basolateral direction measured in **a** fluorescent mode by NTA (F-NTA) and **c** scatter mode (S-NTA). **b**, **d** Recovered amount of particles/sEVs in both compartments at the end of the experiment as a percentage of the initial 10^9^ sEVs containing stock solution. Effect of 0%, 0.25%, 0.5% and 1.0% BSA on particle/sEV recovery after apical sEV application calculated in **b** fluorescent mode by NTA (F-NTA) and **d** scatter mode (S-NTA). **a**–**d** Mean ± SEM, n = 4–6, N = 2–3. Data were analysed with Grubb’s test and by one-way ANOVA followed by Holm-Šidak post-test. *p < 0.05, **p < 0.01, ***p < 0.001

To estimate, and where applicable, reduce the impact of experimental factors on sEV permeation, like pore size of the membranes and surface coatings, blank (cell-free) inserts with different pore sizes, surface (pre-)coatings, and the presence of 0–1% BSA or serum in the assay media were tested. Due to the presence of particles in any kind of cell culture media and to avoid the (well-known) risk of artefacts introduced by the use of lipid dyes [15, 75], GFP-positive vesicles isolated from HEK293-GFP-GPI cells were applied. Pilot experiments indicated that the collagen IV/fibronectin coating, which is essential for optimal conditions in hCMEC/D3-based BBB models (Figure S4), negatively affected sEV permeation across inserts in a concentration dependent manner: Whereas a apical-to-basolateral permeation ratio of 8.53% ± 7.11% was observed across uncoated inserts, collagen IV/fibronectin-coatings reduced permeation in a concentration-dependent manner down to 1.73% ± 0.42% to 4.47% ± 1.07% (Figure S4). Hence to prevent the adhesion of sEVs to matrix proteins (and hydrophobic surfaces), BSA was tested as a blocking- and carrier protein, in combination with a comparison of membranes with pore sizes between 0.4 and 3.0 µm (Fig. 2).

NTA measurements were conducted in fluorescent mode to specifically detect the fluorescently labelled sEVs, whereas measurements in scatter mode provided information about all detectable particle types in the samples (stained and unstained particles). The obtained NTA data revealed that both parameters, pore size and the concentration of BSA in the assay medium, independently affected sEV permeation. Irrespective of the presence of BSA, higher permeation rates of fluorescent vesicles were observed across 1.0 and 3.0 µm membranes (fluo: 6.9%–8.9%) compared to membranes with 0.4 µm pores (fluo: 1.2%), which might pose a physical obstacle for sEV diffusion (Fig. 2a). Secondly, BSA increased the recovery of sEVs (i.e. the percentage of applied fluorescent sEVs which could be retrieved in the apical and basolateral compartment after 24 h) (Fig. 2b). Total particle permeation and recovery (measured in NTA scatter mode) exhibited a concentration dependent BSA effect but given that this was not fully consistent compared to the fluorescent sEVs, it was assumed that BSA also contributed to the overall particle background in the assay (Fig. 2c, d).

In summary, three technical parameters influencing sEV permeation through porous membranes were identified: (1) pores with a diameter > 1.0 µm facilitated higher permeation, (2) addition of BSA as a carrier protein improves sEV permeation and recovery, and (3) sEVs bind to collagen IV/fibronectin matrices.

In the subsequent cell culture experiments inserts with 1.0 µm pores and assay media containing 1% BSA in the basolateral compartment were used to enhance sEV permeation and recovery in the receiver compartment.

### Endothelial cell layers with higher paracellular barrier integrity took up less DU145 sEVs

**Fig. 3:**
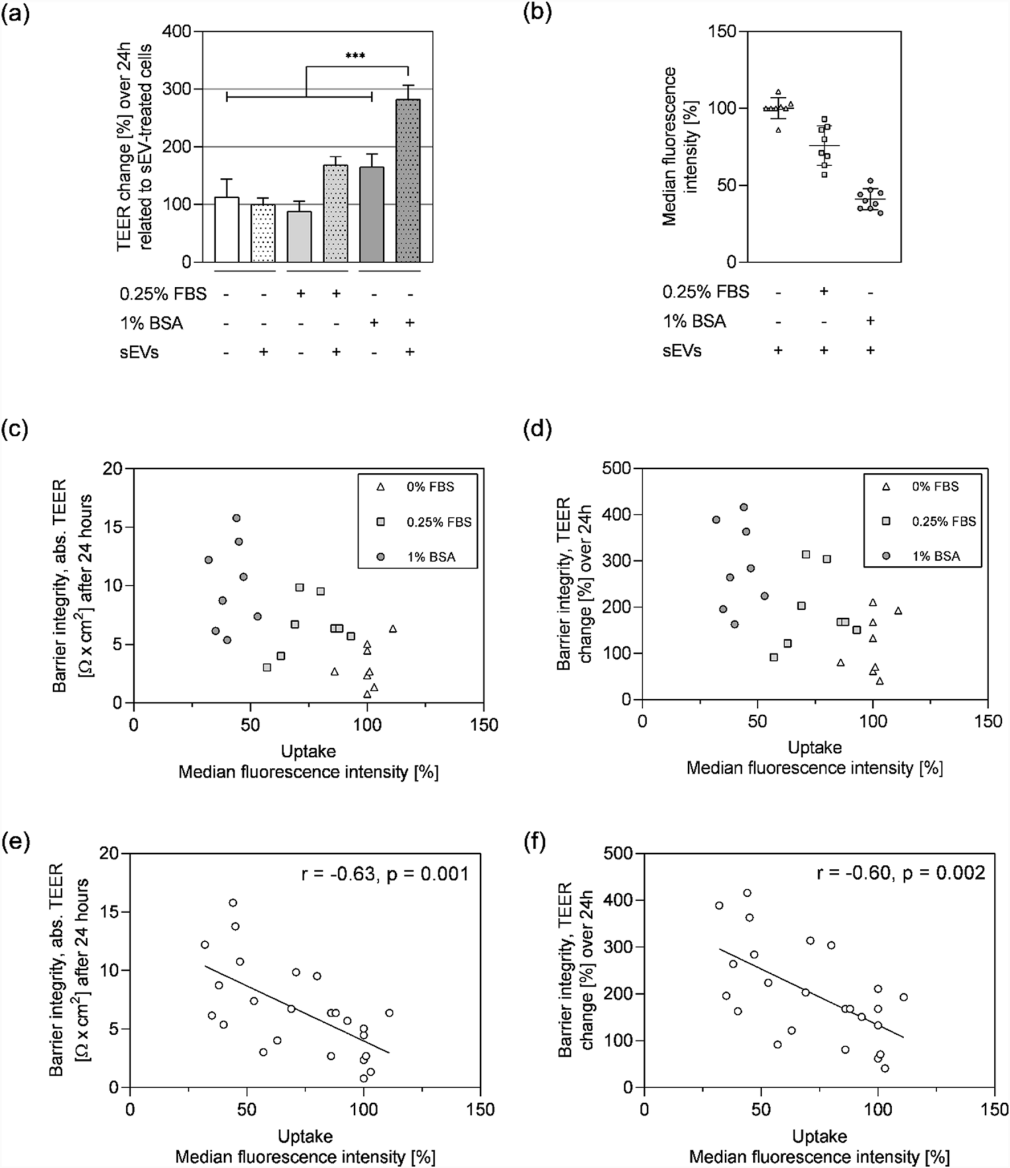
Small EV uptake is negatively correlated with barrier integrity. Effects of 5 × 10^9^ DU145 sEVs (+ sEVs) in different media on **a** TEER and **b** sEV uptake by BBB cells. **a** Changes in TEER after 24 h, normalised to 0% FBS condition. Where no sEVs were added (- sEVs) corresponding labelled medium control was added. **b** Uptake assessed by flow cytometry of CTO labelled sEVs. Per cent of cells that have uptaken CTO labelled DU145 sEVs was normalised to medium labelling control. Inverse correlation between **c**, **e** absolute barrier integrity and sEV uptake (Pearson r = − 0.63, p = 0.001) and **d**, **f** barrier integrity change and sEV uptake (Pearson r = − 0.60, p = 0.002). **a**, **b** Mean ± SEM, n = 8–20, N = 3–6. **c**–**f**, n = 24, N = 3. **a**, **b** Data were analysed with Grubb’s test and by one-way ANOVA followed by Holm-Šidak post-test. *p < 0.05, **p < 0.01, ***p < 0.001. **e**, **f** Linear regression line is shown with Pearson correlation coefficient (r) and significance level indicated at p < 0.05; homoscedasticity as prerequisite for linear regression analysis was confirmed for the data set applying residual plots

Uptake, effects on brain endothelial barrier integrity and permeation of CTO-labelled DU145 sEVs were compared in the hCMEC/D3-based BBB model under three conditions: (1) serum-free media, (2) media containing 0.25% FBS and (3) basolateral media supplemented with 1% BSA. Respective media labelling control samples served as negative controls (Figure S3 and described in detail in the methods section). Interestingly, 24 h after addition of 1% BSA containing basolateral medium, the brain endothelial barrier integrity (quantified by TEER) increased to 165.2% ± 22.4% (p < 0.0001) as compared to serum-free (112.9% ± 31.2%) and 0.25% FBS-containing media (88.1% ± 17.3%). The presence of 5 × 10^9^ DU145 sEVs per mL in combination with 1% BSA in the basolateral compartment, increased the TEER further, up to threefold (282% ± 24.7%; p < 0.0001), as compared to added sEVs to serum-free medium (100% ± 11.1%). A similar effect was observed when DU145 sEVs were added in the presence of 0.25% FBS (increase up to 168.4% ± 14.4%; p = 0.0711) (Fig. 3a). In conclusion, 1% BSA in the basolateral compartment increased TEER values. In this setup, DU145 sEVs did not affect TEER, unless they were added together with a carrier, i.e. BSA or FBS.

Next, the uptake of sEVs by hCMEC/D3 cells was investigated by flow cytometry under above-described conditions. In contrast to the increased TEER values observed upon addition of BSA or FBS together with DU145 sEVs, the uptake of (CTO-labelled) sEVs significantly decreased (1% BSA: 58.88% ± 7.04%, 0.25% FBS: 73.13% ± 8.51%, serum-free: 100% ± 5.17%; p < 0.0001 between 1% BSA and serum-free, and p = 0.002 between 0.25% FBS and serum-free) (Fig. 3b). A significant inverse correlation was observed between barrier tightness (given by TEER) and the uptake of sEVs by hCMEC/D3 cells (Pearson r = − 0.63; p = 0.001) (Fig. 3c), as well as between barrier integrity change in 24 h and sEV uptake (Pearson r = − 0.60; p = 0.002) (Fig. 3d). Interestingly, the recovery of fluorescent sEVs in these cell-based experiments was substantially lower compared to blank insert experiments (Fig. 2), with recovery rates ranging from 21.24 to 27.73% (Figure S5a)—demonstrating the highest percentage when treated with BSA. In summary, when applied with BSA or FBS, DU145-derived sEVs increased the barrier tightness of hCMEC/D3 cell layers, which in turn led to a reduced cellular uptake of the same sEVs. Furthermore, BSA acted as an additional, sEV-independent barrier-stabilizing agent.

### Effects of PCa sEVs on barrier integrity and sEV uptake under inflammatory conditions

Systemic inflammation is common in PCa patients and is correlated with a worse prognosis [99]. In order to mimic inflammatory milieu in the BBB model setup, a previously tested cytokine cocktail of TNF-α, IL-1β and IFN-γ (10 ng/mL each) was added for 24 h [42] together with the sEVs. In addition, sEVs derived from the less aggressive LNCaP PCa cell line were compared to DU145 sEVs at same concentrations (5 × 10^9^ sEVs/mL), to detect potential divergent effects arising from their different cellular origin. Induction of inflammatory conditions by the cytokine cocktail, but not by the treatment with DU145 or LNCaP sEVs, was revealed by an upregulation of gene transcripts involved in inflammatory pathways (Figure S7; VCAM1, ICAM1, RELA, TRAF2, JAK1).

**Fig. 4:**
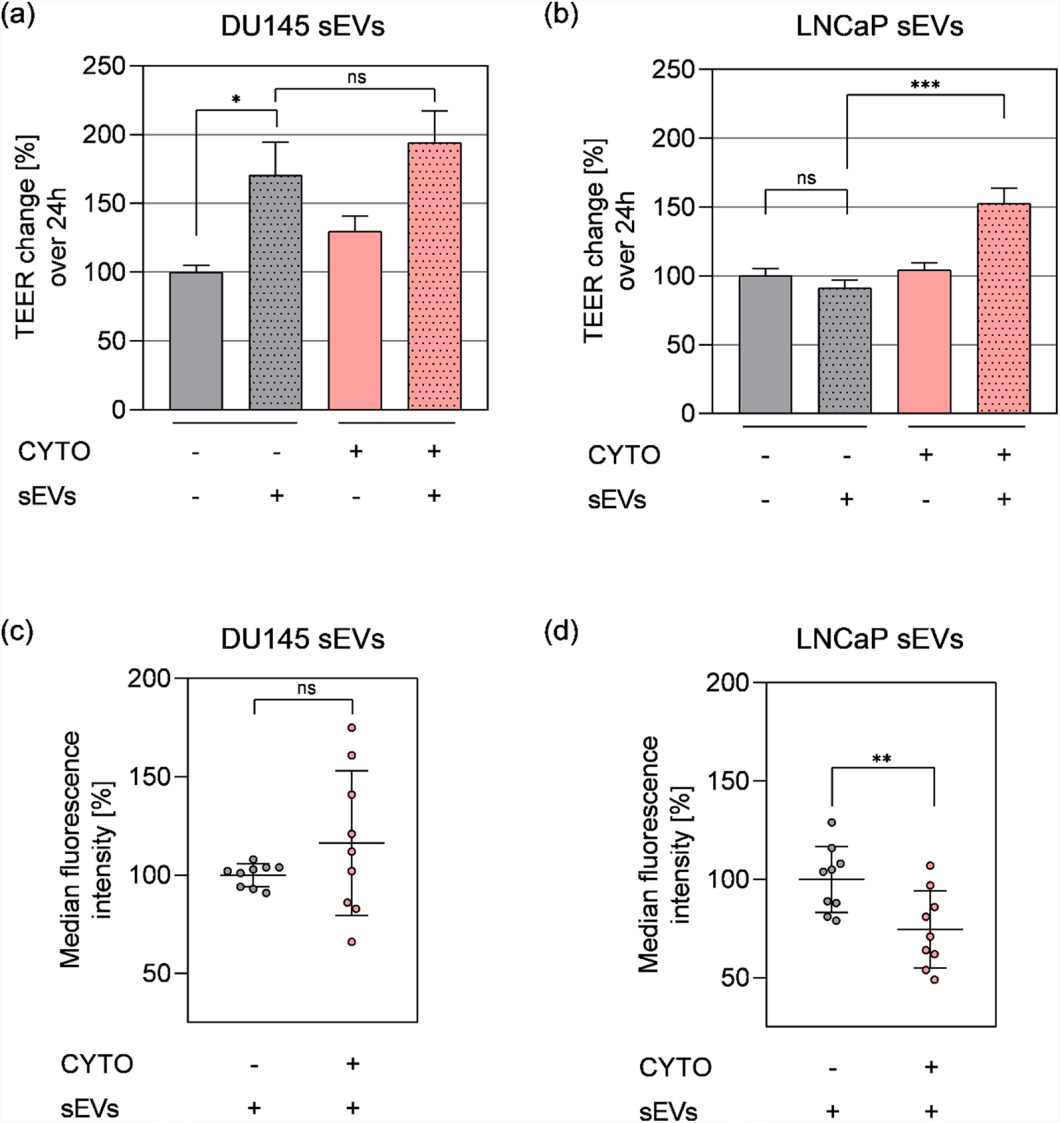
Small EV interactions with hCMEC/D3 are dependent on sEV cell origin. **a**, **b** TEER change after addition of cytokine cocktail (CYTO; 10 ng/ml of TNFα, IL-1β, IFN-γ) and/or 5 × 10^9^ sEVs (+ sEVs) derived from DU145 (**a**) or LNCaP (**b**) in the apical compartments after 24 h incubation. Where no sEVs were added (−sEVs) corresponding labelled medium control was added. **c**, **d** Uptake assessed by flow cytometry of CTO + labelled sEVs. Per cent of cells that have uptaken CTO labelled DU145 (**c**) or LNCaP (**d**) sEVs was normalised to labelled medium control. In all experiments, media used was apically serum-free and had 1% BSA basolaterally. **a**, **b** Mean ± SEM, n = 8–20, N = 3. **c**, **d** Mean ± SEM, n = 9, N = 3. Data were analysed with Grubb’s test and by (**a**, **b**) one-way ANOVA followed by Holm-Šidak post-test or (**c**, **d**) student’s T-test. p > 0.05, ns, non-significant, *p < 0.05, **p < 0.01, ***p < 0.001

Consistent with previous results, DU145 sEVs increased TEER values (170.6% ± 51.3%) as compared to respective media controls (100% ± 10.3%; p = 0.0384) (Fig. 4a), while LNCaP sEVs did not affect the barrier integrity (91.06% ± 12.4%) compared to media controls (100% ± 11.2%; p = 0.6186) (Fig. 4b). Interestingly, the barrier integrity remained unaffected upon cytokine treatments (Fig. 4a, b: DU145 − sEV + CYTO: 129.9% ± 22.9%; p = 0.4362 vs − CYTO; and LNCaP − sEV + CYTO 104.2% ± 11.8%; p = 0.6911 vs -CYTO). Treatment with DU145 sEVs exhibited no additive effect on TEER under inflammatory conditions (Fig. 4a; 194.1% ± 49.7%, as compared to the control: p = 0.4362), whereas the addition of LNCaP sEVs resulted in a significant increase by 61.7% in the presence of cytokines (152.8% ± 23.2%), as compared to the non-treated counterparts (91.1% ± 24.8%; p < 0.0001) (Fig. 4b). The uptake of sEVs, neither from DU145 (Fig. 4c; cytokine treated 134.4% ± 18.8% vs. control 100% ± 1.9%; p = 0.087) nor LNCaP (Fig. 4d; cytokine treated 87.2% ± 13.1% vs. control 100% ± 10%; p = 0.45) cells, was changed significantly under inflammatory conditions. Recovery of fluorescent sEVs under inflammatory conditions ranged from 27.72 to 39.98%, with no significant differences observed between cytokine-treated and control conditions for either DU145 or LNCaP sEVs (Figure S5b).

Besides upregulation and activation of inflammatory pathways (Figure S7), cells were reported to respond to inflammatory cytokine treatment with an increased sEV release [67, 70, 100]. NTA measurements of apical particles under inflammatory conditions revealed a significant increase of sEV-like particles released by the hCMEC/D3 cells: inflammatory cytokine treatment alone (Figure S8: media control; non-treated: 1.6 × 10^9^ sEV-like particles/mL vs. cytokine treated: 3.3 × 10^9^ sEV-like particles/mL, p < 0.001). Treatments with DU145 sEV did not obviously affect this inflammatory cytokine treatment-induced particle release (Figure S8: non-treated: 5.5 × 10^9^ sEV-like particles/mL vs. cytokine treated: 7.4 × 10^9^ sEV-like particles/mL, p < 0.001).

In summary, both DU145 and LNCaP sEVs affect the function of hCMEC/D3 brain endothelial cells in distinct manners. Whereas DU145 sEVs led to an increase of TEER, LNCaP sEVs induced a similar effect only under inflammatory conditions. Furthermore, uptake and release of sEVs were found to be influenced by parameters like the presence of BSA or FBS, barrier integrity and inflammatory status, indicating a complex interplay between cellular, biophysical, and molecular factors in this BBB model.

### Changes in mRNA expression of hCMEC/D3 cells upon sEV and inflammatory treatments

**Fig. 5:**
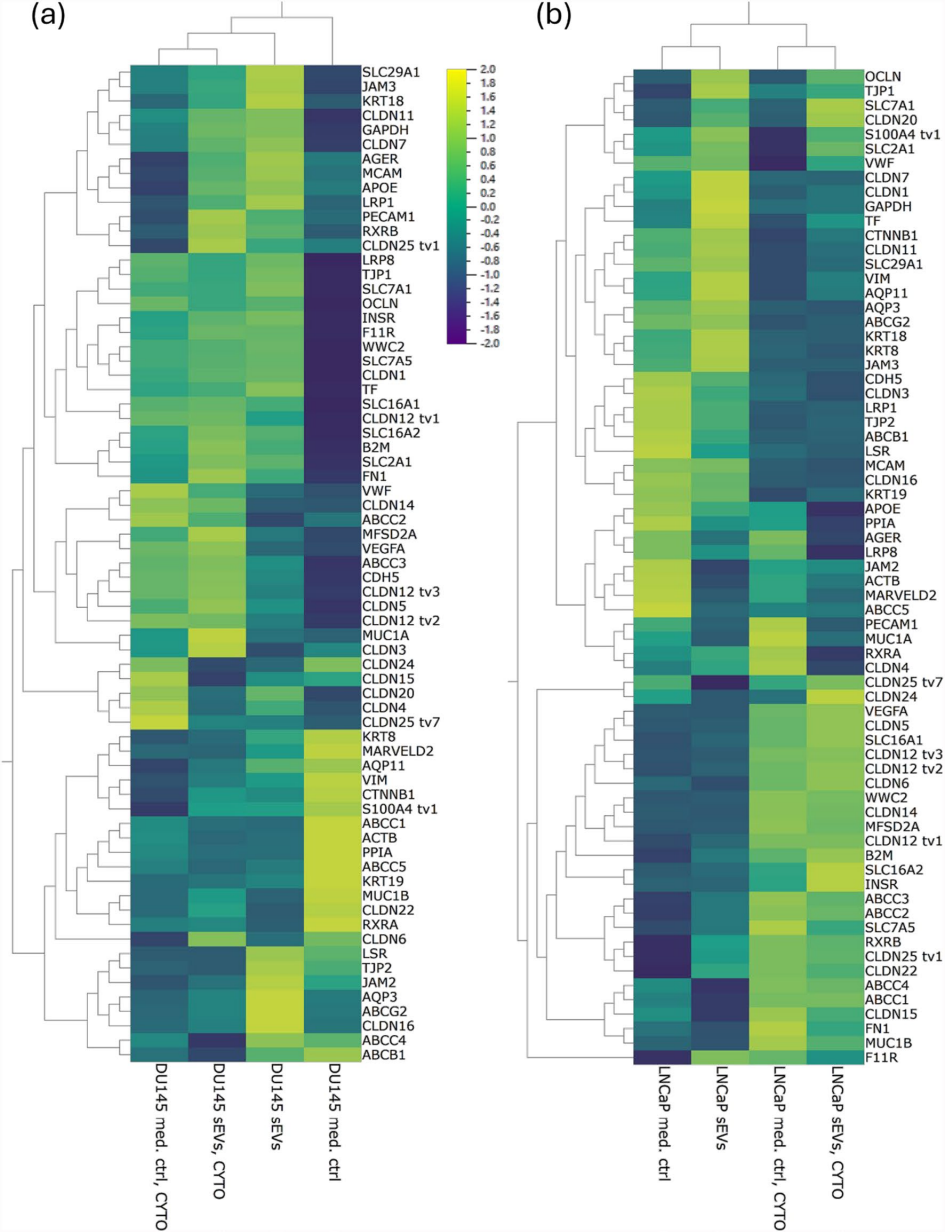
Heatmap and hierarchical clustering of mRNA expression from hCMEC/D3 cells treated with cytokine cocktail (CYTO) and/or sEVs derived from PCa cell lines: **a** DU145 and **b** LNCaP cells or treated with their respective medium controls (MC). Threshold cycle (Ct) values were normalised to the respective sEV treatment. Hierarchical clustering was performed using an unsupervised approach to explore and visualize how BBB-related genes group across experimental conditions, with the primary aim of differentiating the effects of inflammatory cytokine treatment versus sEV-induced changes and identifying overarching expression patterns within the dataset. X-fold expression of the mean values of three independent experiments are displayed using the colour scale “viridis”, with 2 (yellow) indicating a high expression and − 2 (purple) indicating a low expression value. The colour code legend is added and formatted in log-scale. Original x-fold gene expression data are available in the supplementary files (Table S6 and S7) for reference. Mean ± SD, n = 3, N = 3

To capture the potential impact of sEVs and/or cytokine treatment on the transcriptional level of BBB-relevant genes, i.a. encoding junctional proteins, BBB markers, and transporter proteins and receptors, an established high-throughput qPCR chip analysis was conducted. Consistent with the observed effects on hCMEC/D3 barrier integrity (Fig. 4a), DU145 sEVs triggered a significant upregulation of claudin-1 (Table S6; CLDN1, p = 0.0372) in comparison to the respective media controls (Fig. 5a). Furthermore, fibronectin was significantly upregulated when DU145 sEVs were added under inflammatory conditions as compared to media controls (Table S6; FN1, p = 0.0258). LNCaP sEVs, but not DU145 sEVs, led to a significant downregulation of VE-cadherin upon inflammatory cytokine treatment, as compared to the non-inflamed media controls (Table S7; CDH5, p = 0.0372). Hierarchical clustering of the expression data indicated that in samples treated with LNCaP sEVs, inflammatory cytokine treatment was the primary clustering parameter, whereas in samples treated with DU145 sEVs, the sEVs were the predominant factor (Fig. 5)—concordantly with the overall stronger effects of DU145 sEVs observed in previous experiments.

## Discussion

In this study, the effects of PCa-derived sEVs with different metastatic potential from two cell lines on a human BBB in vitro model were investigated. Previous experiments have indicated that technical parameters in in vitro cell culture insert systems may restrict the permeation of sEVs, hence an extensive optimization of the established setup had to be performed.

Drug transport studies across the BBB are typically done with cell culture membrane inserts with 0.4 µm pores, in order to achieve higher TEER values, in particular in models based on the hCMEC/D3 cell line [6, 34–37, 65–69, 81]. In case of sEVs, first tests showed, that sEVs > 100 nm cannot freely diffuse across such cell culture membrane inserts, and even after 24 h incubation time, a concentration equilibrium between apical and basolateral compartment was not reached (Fig. 2). Thus, as a first important suggestion for sEV transport studies, pore sizes bigger than 0.4 µm should be used. Secondly, the insert surface coating matrix commonly used for BBB models, composed of collagen IV and fibronectin, represented a barrier and/or trap for sEVs (Figure S4), most likely due to the interaction of e.g. integrins present on sEVs with extracellular matrix proteins [3, 82]. As this matrix is important to achieve high quality BBB models [69], BSA was added to the system to block or at least minimize interactions between sEVs and matrix proteins, as well as unspecific adhesion to hydrophobic (plastic) surfaces [19]. Recent reports on protein corona formation around sEVs [86, 97] may provide an additional explanation for the observed beneficial effect of BSA in the presented, optimized model (Fig. 2).

The final, optimized setup used throughout this study, combined following conditions to allow increased sEV permeation (1.0 µm pores) and improved recovery (addition of 1% BSA), and to establish a cell layer with BBB properties (surface coating with collagen IV/fibronectin). In order to avoid potential known artefacts arising from labelling sEVs with lipid dyes, such as labelling of non-vesicular particles and formation of dye aggregates [13, 75], in the optimization phase, GFP-positive sEVs produced by HEK293-GFP-GPI cells were applied. Indeed, when comparing scatter and fluorescent particle measurements, a small contribution of media particles and minor background caused by BSA addition could be observed (Fig. 2, Figure S6). Further experiments were performed with non-modified (untransfected) PCa cells, hence the membrane-permeant stain CellTracker™ Orange (CTO) was used to detect PCa sEVs. CTO is acquiring fluorescence only upon cleavage by intravesicular esterase activity [15], and such labelled vesicles exhibited very similar properties in the test setup, as compared to GFP-sEVs (Figure S6). In order to consider possible inherent signal background due to sEV purification and fluorescent labelling procedures, media labelling controls were included in each experiment (Figure S3) and documentation of labelling efficiencies (Table S5) were crucial for sound interpretation of the obtained results. However, it cannot be excluded that application of sEVs derived from PCa cell lines for blank experiments might lead to deviating results compared to sEVs from non-malignant sources, such as HEK293. Additionally, while our study focused on apical sEV application to model the physiologically relevant blood-to-brain (apical-to-basolateral) route, we acknowledge that we did not systematically investigate potential polarized effects of sEVs on BBB integrity when applied to different compartments of the BBB model.

The BBB represents an extremely tight biological barrier, preventing the fast metastasis of primary tumours originating from peripheral organs. On the other hand, tumours forming inside the brain, are largely protected by the BBB from attacks of the immune system and therapeutic compounds. Despite its strong barrier function, 10–30% of cancer patients suffer brain metastases, usually coinciding with the terminal phase of the disease. Small EVs have been implicated in the early phases of brain metastasis by disturbing BBB functions and facilitating pre-metastatic niche formation. In order to investigate these potentially adverse effects of sEVs in the context of prostate cancer, two well-characterized PCa cell lines, DU145 and LNCaP, were selected for this study.

BBB in vitro models are highly susceptible to culturing conditions, e.g. media conditions, cytokine treatments, and co-cultures with other cell types, which modulate the endothelial barrier properties. In the current study, basolateral BSA supplementation was identified as a general barrier stabilizing factor (Fig. 3a). Previous studies have shown correlations between sEVs under different pathological and inflammatory conditions, and an increased endothelial permeability in vitro [11]. Cancer derived sEVs, from colorectal carcinoma [106], hepatocellular carcinoma [20], and brain cancers [85, 88], were reported to disrupt endothelial barrier integrity by downregulating tight- and adherens junction proteins, and disrupting the actin cytoskeleton. Notably, sEVs isolated from DU145 had the opposite effect on hCMEC/D3 barrier integrity in the presented study: TEER was stabilized or even increased in the presence of DU145 sEVs, in particular when BSA (or serum) was present in the medium (Figs. 3a and 4a), whereas LNCaP sEVs did not exert a significant impact on barrier integrity. It remained unknown, whether this difference was an intrinsic property of the applied sEVs or resulting from the specific combination of hCMEC/D3, the assay- and DU145/LNCaP cell culture conditions. Additionally, it should be considered that the phenotypic characteristics of cancer cell lines can be influenced by culture conditions, which may affect sEV composition and functional properties. To strengthen the findings regarding cancer aggressiveness (especially in the context of PCa-derived sEVs) and effects on BBB barrier integrity, future studies with an expanded panel of cell lines would be needed.

A possible explanation for the reason why EVs from the more aggressive cancer cell line increased BBB integrity could be that the cargo of DU145-derived sEVs is more potent and may include regulatory molecules (such as specific RNAs, proteins, or lipids) that transiently activate protective or compensatory mechanisms in endothelial cells, such as the upregulation of tight junction components or anti-inflammatory pathways. Although, at first glance this seems unexpected, particularly since tumor-derived sEVs and even tumor-cell signalling in general might be expected to compromise the BBB in vitro [40, 85, 88, 105], however, clinical data revealed that even in the most aggressive tumors such as glioblastoma, the BBB is not completely compromised and can even be more selective in regard to drug transport to the brain [71]. In addition, the BBB was also reported to be differentially preserved depending on a specific metastatic breast cancer type. For example, triple negative basal-type breast cancers often led to BBB disruption, whereas HER2/neu-positive breast cancer—that was more likely to metastasize the brain—tended to preserve the BBB more often [103]. Another aspect in this regard could be that the inverse correlation between sEV uptake and TEER might also reflect differences in sEV surface markers or lipid composition that influence internalization pathways. It could be possible that higher sEV uptake, as observed with the less aggressive cell line, triggered stress responses or barrier disruption, whereas lower uptake from DU145 sEVs might spare the cells from such responses or deliver cargo through more selective mechanisms that are currently unknown. Overall, further studies are necessary to elucidate the mechanisms and provide finally a more conclusive explanation.

Interestingly, higher paracellular barrier tightness correlated with a reduced uptake of DU145 sEVs by the brain endothelial cells (Figs. 3c, d). Other studies have demonstrated that a tighter BBB correlated with reduced transcytosis rates, which is one of the mechanisms suggested for sEV uptake and transport across the BBB [12, 54]. Whereas several reports using in vitro and in vivo models postulated EV transport across the BBB, in the presented study sEV permeation across the hCMEC/D3 cell layer was not detectable, neither by means of NTA nor a highly sensitive, novel EV detection method, EVQuant [27] (data not shown). It is important to note, that—to our best knowledge—none of the reported experimental setups could confirm the transport of intact single sEVs across a BBB model, but relied on the detection of labelled cargoes or membranes (fluorescent, radioactive or enzymatic) [12, 33, 51, 54, 85]. In general, the BBB is considered to exhibit extremely low transcytosis rates [2], concordant with the results obtained in this study.

Another well-known factor connected to BBB integrity and important during tumour progression is inflammation [26, 60, 92, 99]. Endothelial cells are activated by pro-inflammatory cytokines [41] and several in vitro studies have shown that different cytokines mimicking inflammatory milieu, modified BBB properties and led to increased permeability [10, 44, 57, 92, 107]. A hallmark of endothelial pro-inflammatory response is the upregulation of adhesion molecules such as intercellular adhesion molecule-1 (ICAM-1) [18] and vascular cell adhesion molecule-1 (VCAM-1) [80]. Both, ICAM-1 and VCAM-1 expression were found upregulated following a 24 h treatment with a pro-inflammatory cytokine mix (Figure S7), as reported before [11, 58, 92, 94]. Despite this inflammatory response, TEER remained stable in the presented model (Fig. 4), possibly due to the barrier-strengthening action of IFN-γ [56] and beneficial effects exerted by the addition of BSA to the basolateral media. Additional treatment with DU145 or LNCaP sEVs had no effect on the expression level of the tested inflammatory genes (Figure S7), nor was the sEV uptake changed by the applied cytokines (Fig. 4). Notably, hCMEC/D3 cells responded to cytokine treatments with an increased release of sEV-like particles, which were detected in the apical compartment (Figure S8). A similar effect was reported in hCMEC/D3 cells after TNF-α and IFN-γ treatment [32, 67]. Interestingly, sEVs released by those cytokine-treated hCMEC/D3 cells compromised the barrier integrity when applied to non-treated hCMEC/D3 cells [67], indicating that sEVs can function as a vehicle for inflammatory signals (and other factors, potentially adsorbed to the EVs’ protein corona), which subsequently affect cell–cell contacts in recipient cells. However, it remains unclear whether exposure to PCa cell-derived sEVs induced sEV release from hCMEC/D3 cells; if so, autocrine signaling mechanisms had maybe contributed to the observed phenotypic changes, as previously suggested in the context of inflammatory activation of these brain endothelial cells [67].

Changes in the mRNA expression pattern of hCMEC/D3 cells upon sEV treatment without inflammatory cytokines and under inflammatory conditions were assessed by an in-house established high-throughput qPCR chip covering about 90 barrier-relevant transcripts [23, 43, 62]. Few significantly changed transcripts were detected, in line with the moderate changes observed on cellular and functional level. Interestingly, two targets found upregulated in DU145 sEV treated cells were fibronectin and claudin-1. Their upregulation corresponded to the elevated TEER values observed in the presented model when BSA or FBS were present in the medium (Fig. 3a). Fibronectin has essential functions as an extracellular matrix component at the BBB and in preserving barrier integrity [64, 84, 93]. In addition to DU145 sEVs, fibronectin expression exhibited a similar trend upon treatment with cytokines, consistent with its strong involvement in inflammatory processes and endothelial cell remodelling [9, 46]. Reports on the role of claudin-1 as a tight-junction forming protein at the BBB are ambiguous. Depending on the physiological/pathological conditions and/or the experimental model employed, claudin-1 has been implicated both, in a weakening and enhancement of barrier integrity, indicating that its role may depend on the physiological, cellular and molecular context [28, 59, 65, 76, 108]. In view of its upregulation upon application of DU145 sEV, claudin-1 (as a tight junction forming claudin) may have contributed to an increase of barrier tightness. As observed for fibronectin, claudin-1 expression exhibited a similar trend under inflammatory conditions. In comparison, vesicles derived from the less aggressive LNCaP cells did not induce significant changes in gene expression, with exception of VE-cadherin, which was found downregulated when LNCaP sEVs were applied together with inflammatory cytokines. The same trend was observed under inflammatory cytokine treatment without sEVs present, indicating, together with upregulation of VEGF (irrespective of DU145 or LNCaP sEVs present), that inflammatory cytokine treatment was the predominant factor in this model and potential additive or synergistic effects of DU145 and LNCaP sEVs were below the intrinsic experimental variability. Furthermore, in both, DU145 and LNCaP setups, the transcripts of claudin-14 and major facilitator superfamily domain-containing protein-2a (MFSD2a) were up-, whereas ABCB1 was found downregulated in response to inflammatory cytokines, independent of sEV treatments. ABCB1 mRNA expression has been found downregulated in vivo previously in rat- [24] and mouse models [78]. A downregulation of ABCB1 upon inflammatory cytokines induced by TNF-α and IFN-γ was demonstrated in rodent [104] and human BCECs before [30, 39]. The complex role of MFSD2a in BBB maintenance has not been entirely elucidated yet but involves i.a. a regulatory function of BBB permeability via modulation of transcytosis, linked to pathological and inflammatory cues [17]. Claudin-14 has not been identified as a relevant junctional protein at the BBB so far [72], also the remarkable upregulation of claudin-14 as seen upon inflammatory cytokine treatment in the presented data set, was not reported so far. Based on a previous study indicating a role of claudin-14 in tumor angiogenesis [4], a functional role in endothelial cells might be possible.

## Conclusions

In conclusion, the presented results illustrate that diverse technical and experimental parameters can strongly affect particle permeation across BBB models and should be considered in such setups. Most critical in this view was the choice of the membrane pore size, where 0.4 µm pores drastically restricted sEV diffusion. Supplementation of assay media with 1% BSA reduced the loss of sEVs due to binding to matrix proteins and unspecific adhesion to plastic surfaces—on the other hand, addition of BSA per se increased the TEER values in the employed hCMEC/D3 based model considerably. Both, DU145 and LNCaP sEVs were readily uptaken by hCMEC/D3 cells, but only DU145 sEVs led to an increase of barrier integrity. On the other hand, higher TEER values, correlated with reduced sEV uptake, indicating a regulated uptake mechanism. Transport of sEVs across the cell layer to the basolateral compartment remained below detection limits of NTA and EVQuant. In view of studies postulating sEV transport across the BBB, based on the detection of permeated cargo, the intracellular fate of sEVs in brain endothelial cells remains to be determined. Additionally, the substantially reduced recovery of sEVs in cell-based experiments compared to blank inserts highlights the complex fate of sEVs in cellular environments, involving internalization, adhesion, degradation, and potential cellular processing. Future work focused on developing and employing single sEV tracking across the cell layer in real-time may help to resolve this more precisely. If, and at which rate, intact sEVs are transported through brain endothelial cell layers was not conclusively reported yet.

## Supplementary Information

Supplementary Material 1.

Supplementary Material 2.

## Data Availability

Data is provided within the manuscript or supplementary information files.
