# Supplementary material for: Uptake of DU145 and LNCaP prostate cancer cell line derived extracellular vesicles is inversely correlated with blood–brain barrier integrity in vitro
Source: Fluids Barriers CNS. 2025 Jul 7;22:70. doi: 10.1186/s12987-025-00680-7 (PMC12232752; doi:10.1186/s12987-025-00680-7)
Supplement: Supplementary file 1 — Supplementary Material 1. [file 12987_2025_680_MOESM1_ESM.docx]

Supplementary figures


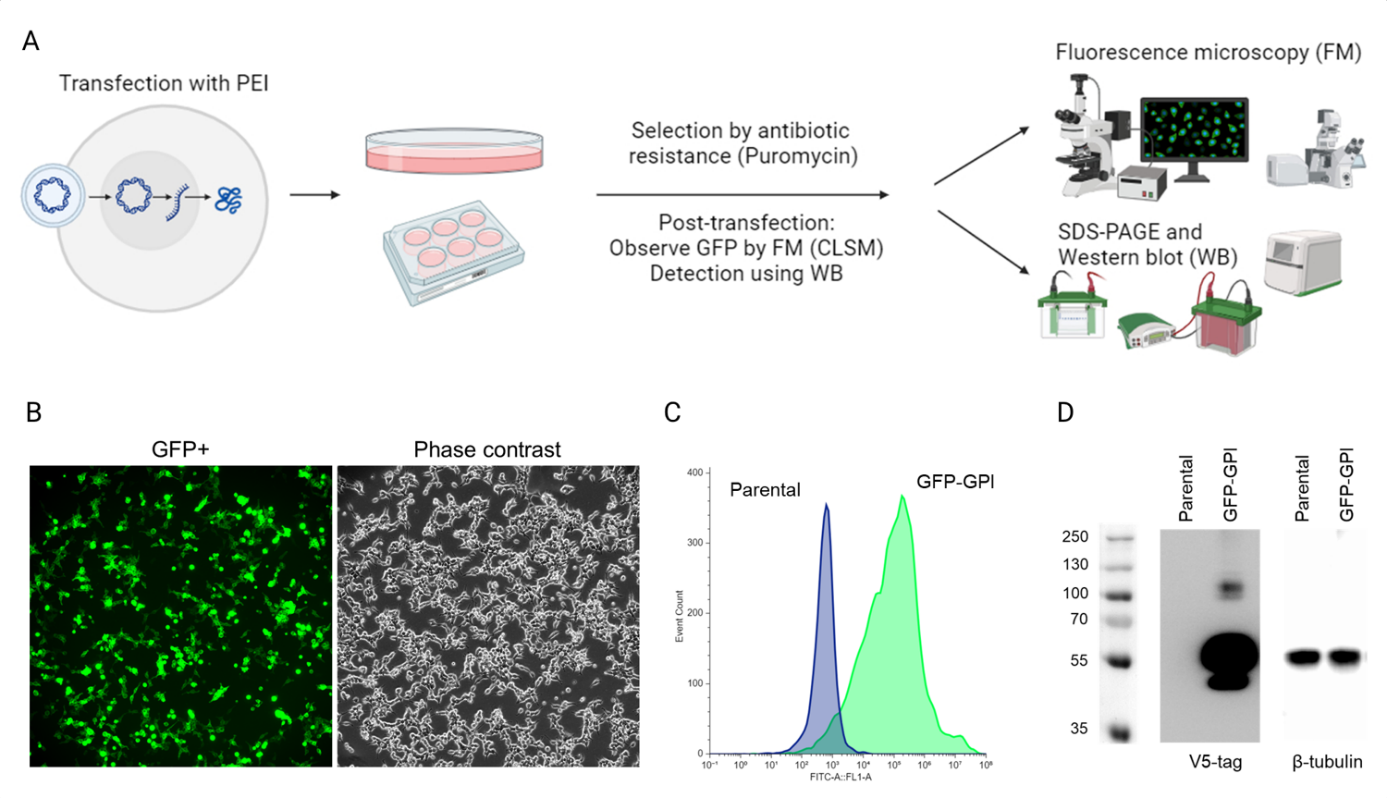


Figure S1: Transfection of HEK293 cells with plasmid GPI-anchored GFP (GFP-GPI) for blank Transwell® experiments. (A) Schematic overview of transfection and the determination of its efficiency. Cells were transfected by plasmid DNA carrying GPI-anchored GFP (GFP-GPI) and puromycin antibiotic resistance gen with PEI agent. Subsequently, the antibiotic-resistant transfected cells were selected from their parental/untransfected neighbours with puromycin to establishing stable transfected pools. Cells were observed by fluorescence microscope (FM) and Western blot (WB). Workflow was created with Biorender.com. (B) Representative images of HEK293 GFP-GPI transfected cells after puromycin selection. Transfected cells were positive for GFP (green). Scale bar = 100 µm. (C) Representative flow cytometric histogram on the transfected GFP+ population. (D) Total cell protein lysates of parental HEK293 and HEK293 expressing GFP-V5-GPI, incubated in growth medium were analysed by WB. V5-tagged recombinant proteins (constructs) were found in the transfected cells. β-actin was used as loading control. 10 µg of protein was loaded per sample.


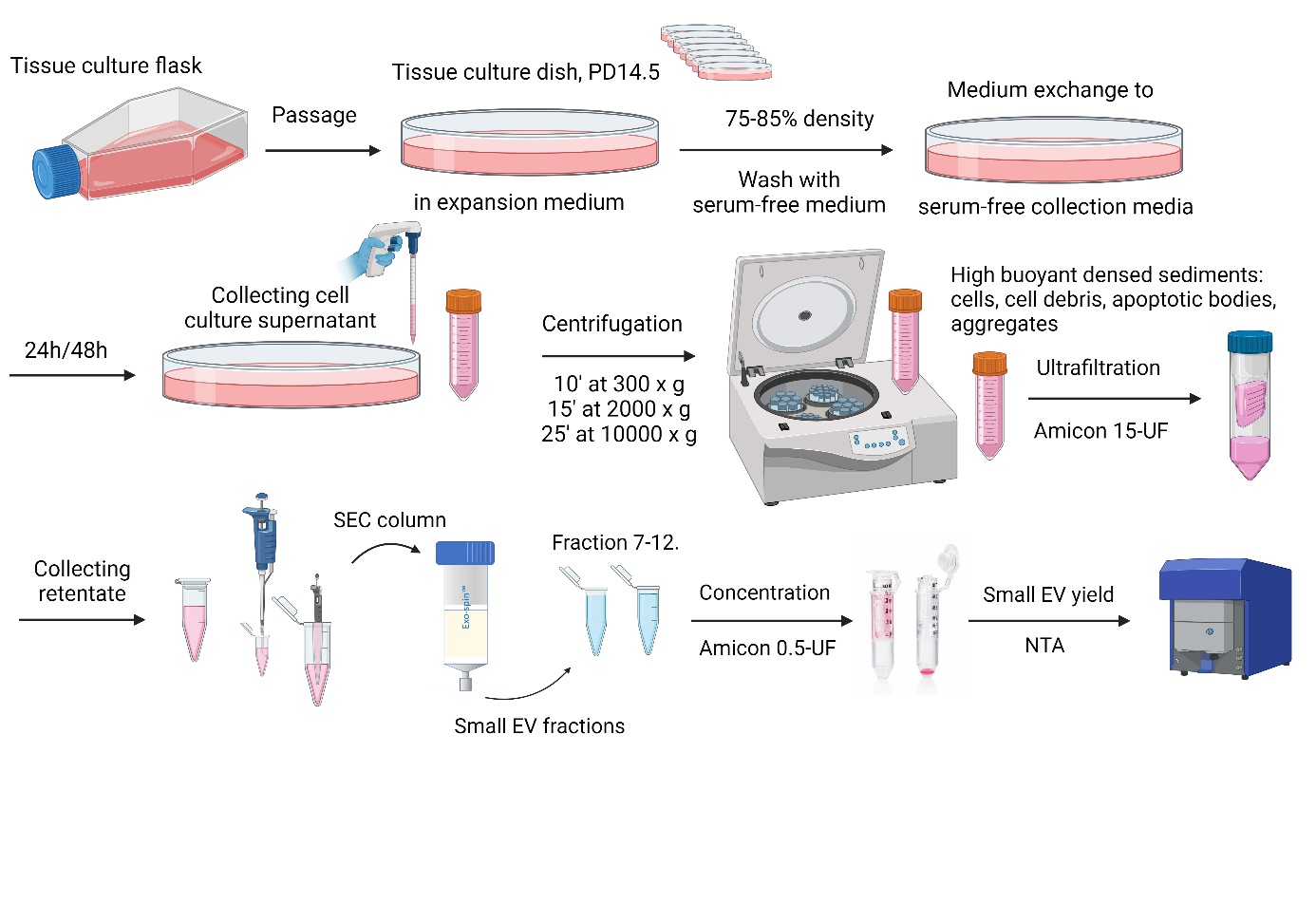
Figure S2: Workflow of small EV enrichment. After maintaining cells in tissue culture flasks, they were cultivated onto large Petri-dishes (PD14.5) to expand them. Having reached a certain density, cells were incubated in foetal bovine serum (FBS)-free conditioning media for 1 or 2 days. After collecting cell culture supernatant, high buoyant dense sediments were depleted, and samples were ultrafiltrated. Small EVs from the retentate of depleted and concentrated cell culture media were purified using size exclusion chromatography (SEC) columns. Small EV fractions were collected and concentrated. Small EV yield was measured on Nanoparticle Tracking Analysis (NTA). The figure was created using Biorender.com.


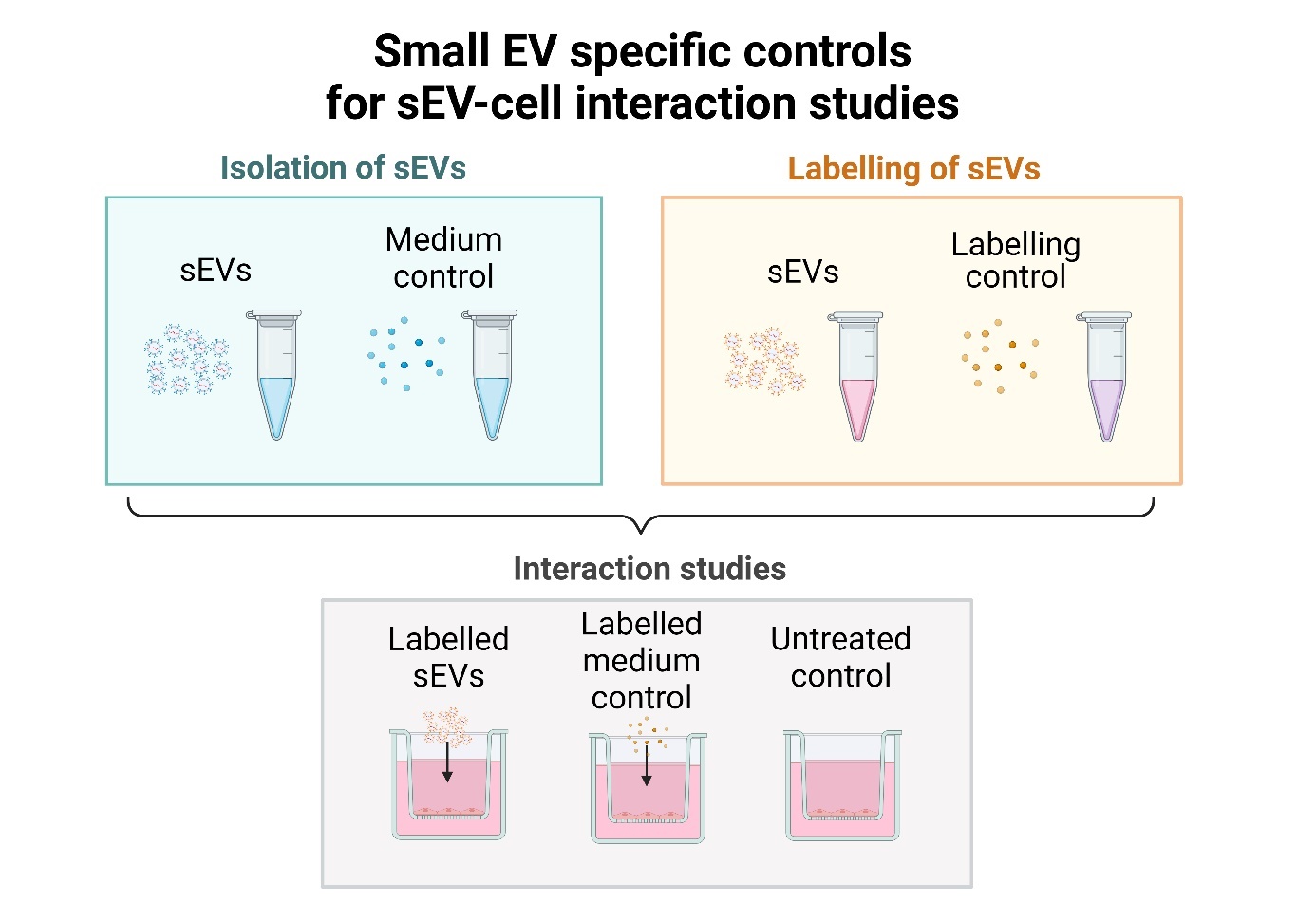
Figure S3: Small EV specific controls for sEV-cell interaction studies. Medium controls consist of serum-free medium-derived particles purified using the same method as cell-derived sEVs described in Figure S2. Labelling controls include Cell Tracker Orange^TM^ (CTO)-labelled, DPBS-based particles. Both labelled medium controls and labelled sEVs undergo purification and labelling processes to study interactions. Therefore, medium controls are essential in all experiments and in their analyses. Untreated controls are important for assessing the barrier functionality and for subtracting the background median fluorescence intensity of the cells regarding the uptake. The figure was created using Biorender.com.


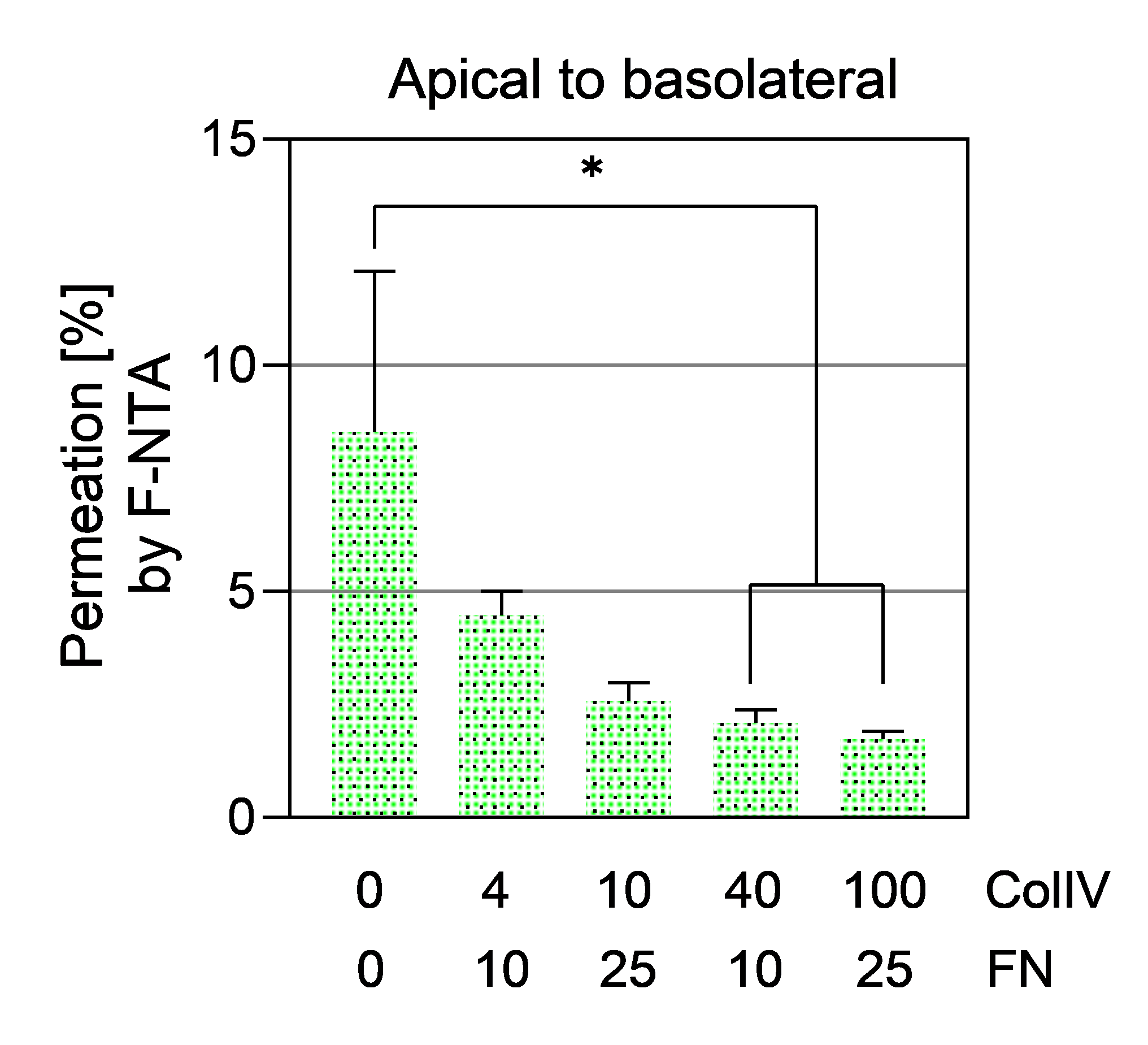


Figure S4: Influence of coating matrix, insert properties, precoating with serum on sEV recovery and permeation with 10^9^ sEVs derived from HEK293 GFP-GPI in different pore size insert models for 24h. Effect of coating matrix on sEV recovery using 0.4 µm pore size inserts, collagen IV (ColIV; µg) and fibronectin (FN; µg) coating mixture and fluorescent sEVs assessed by Fluorescence Nanoparticle Tracking Analysis (F-NTA). Mean ± SEM, n=4-6, N=2. Data were analysed with Grubb’s test and by one-way ANOVA followed by Holm-Šidak post-test. *p < 0.05, **p < 0.01, ***p < 0.001.


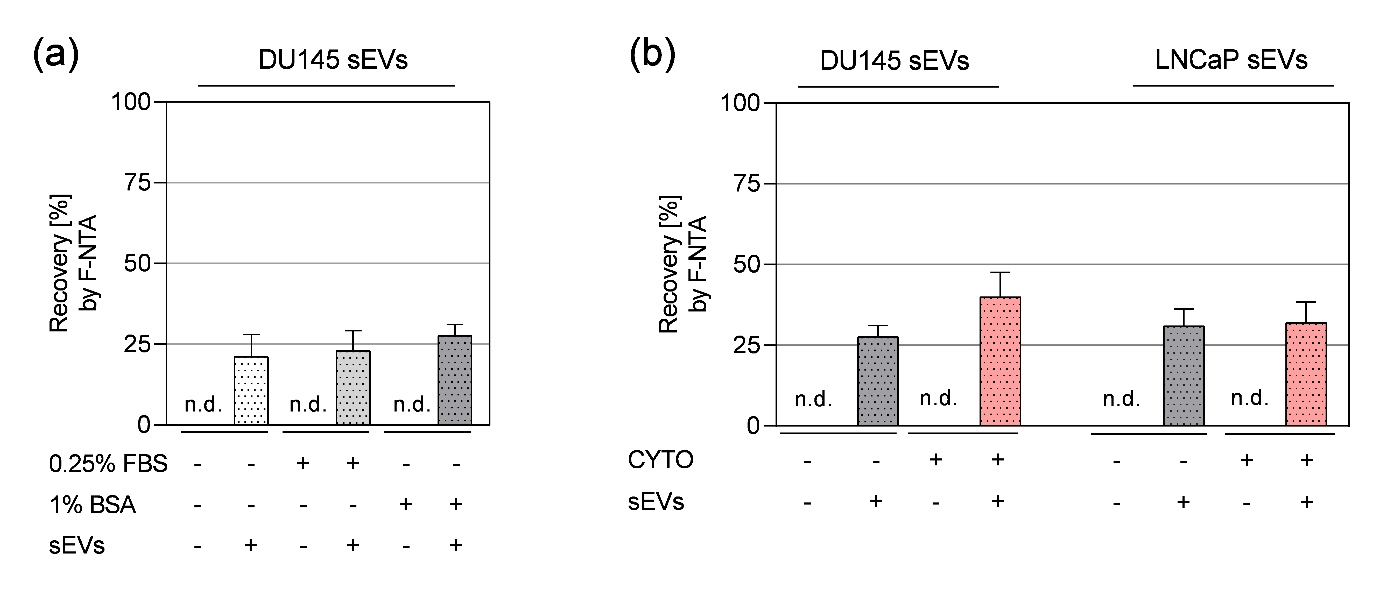


Figure S5: Recovery of fluorescent sEVs in the cell experiments after 24h. Recovered amount of sEVs at the end of the experiment as a percentage of the initial 5 x 10^9^ sEVs containing stock solution. Recovery (a) related to Figure 3 of the manuscript with DU145 sEVs (+ sEVs) or with their corresponding labelled medium control (- sEVs) in different media and (b) related to Figure 4 of the manuscript with co-addition of cytokine cocktail (CYTO; 10 ng/ml of TNFα, IL-1β, IFNγ) and/or 5 x 10^9^ sEVs (+ sEVs) derived from DU145 (left) or LNCaP (right) after 24h incubation. In panel B, media used was apically serum-free and had 1% BSA basolaterally. Mean ± SEM, n=13-27, N=3. Data were analysed with Grubb’s test and either (a) by one-way ANOVA followed by Holm-Šidak post-test or (b) student’s T-test (with p < 0.05 as significant).


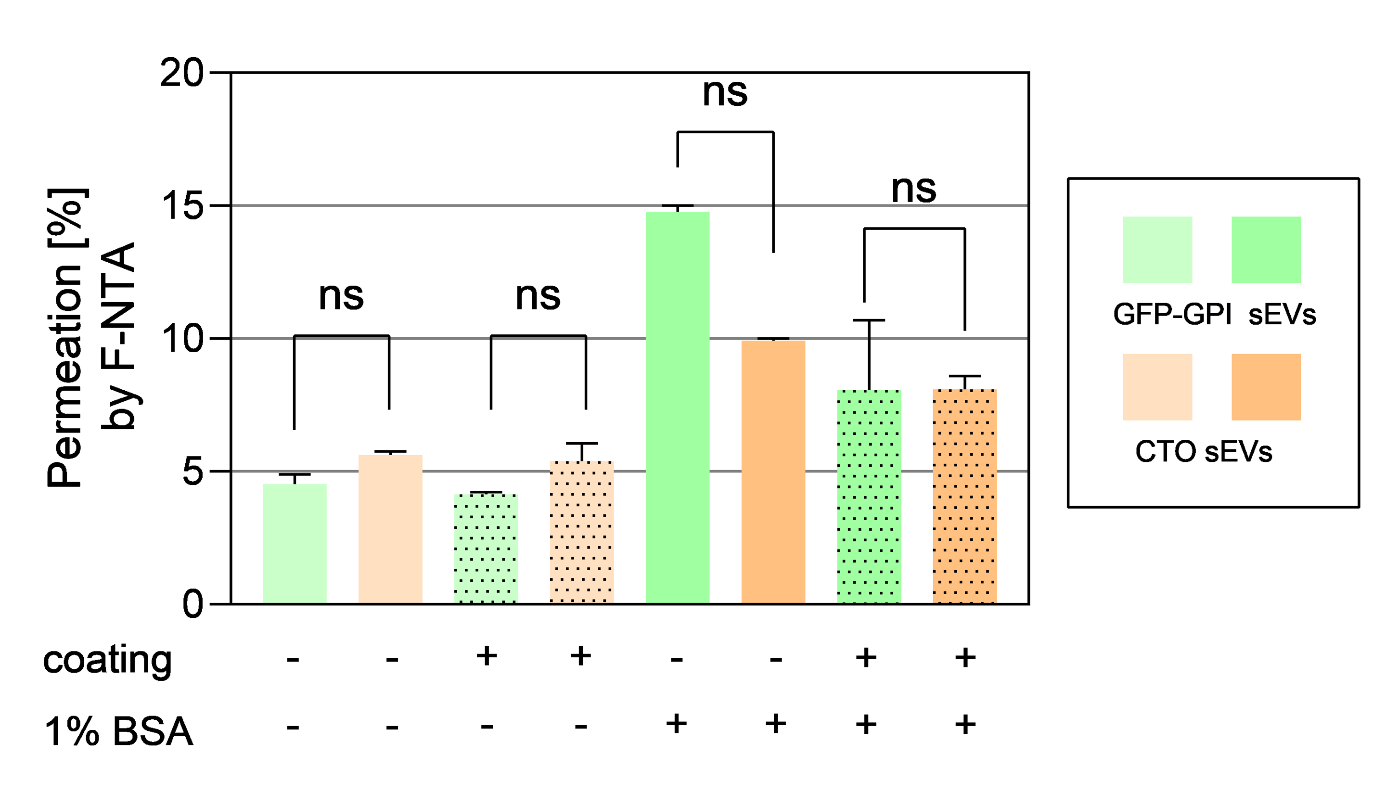


Figure S6: Influence of coating matrix and the presence of 1% carrier protein (BSA) on sEV permeation using HEK293 GFP-GPI-derived and HEK293 CTO-labelled 10^9^ sEVs in 1.0 µm pore size insert model for 24h. Permeated sEVs measured by Fluorescence Nanoparticle Tracking Analysis (F-NTA). Mean ± SEM, n=3. Data were analysed with Grubb’s test and by one-way ANOVA followed by Holm-Šidak post-test. p > 0.05, ns, non-significant.


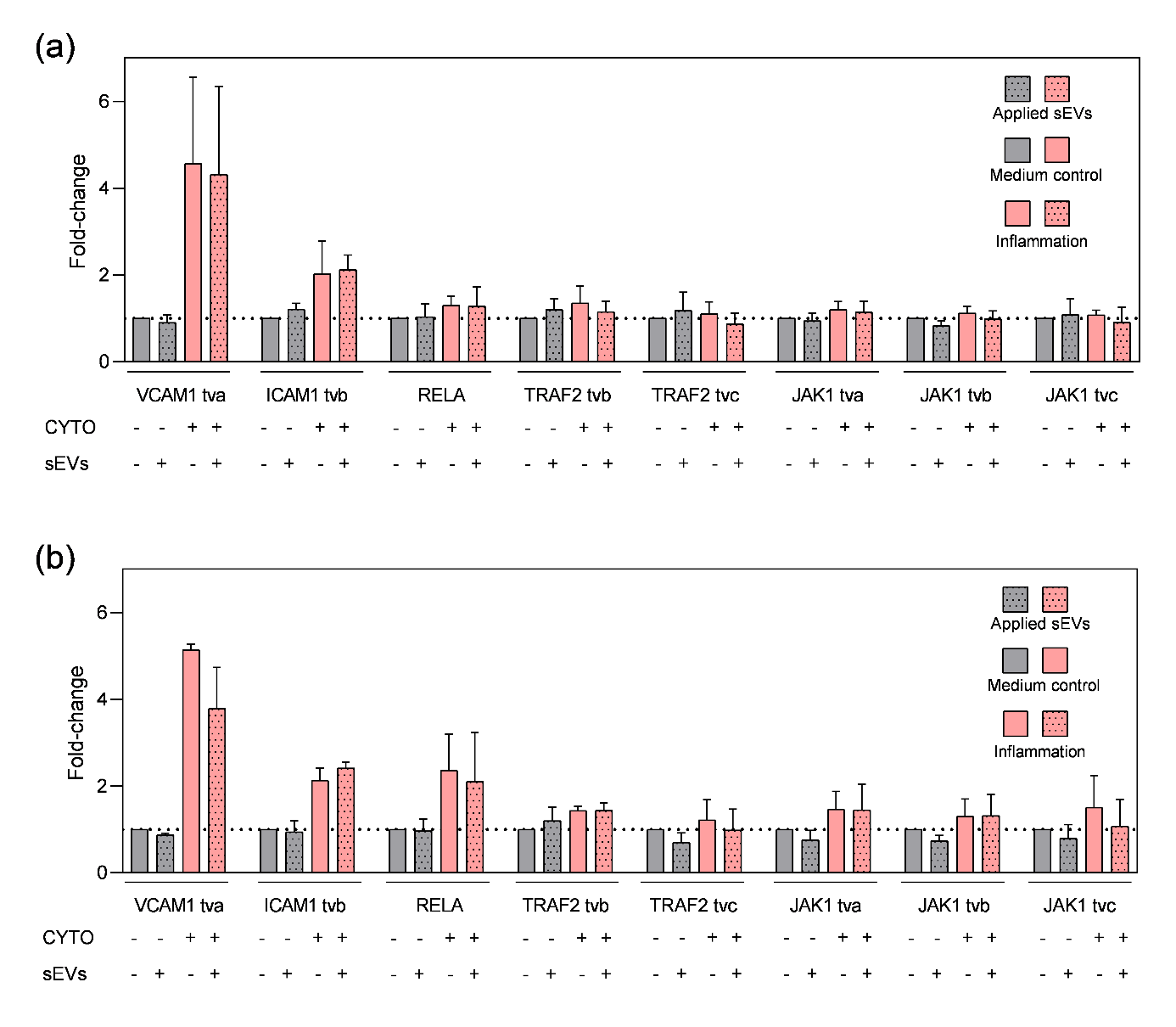


Figure S7: Typical inflammatory targets analysed at mRNA level after treating hCMEC/D3 cells with cytokine cocktail (CYTO) and sEVs (+ sEVs) derived from PCa cell lines for 24h: (a) DU145 and (b) LNCaP cells. Where no sEVs were added (- sEVs) corresponding labelled medium control was added. Threshold cycle (Ct) values were normalised to the endogenous housekeeping genes and relative quantification was performed based on the comparative 2−ΔCt method of different groups to untreated control samples. Mean ± SEM, N=3.


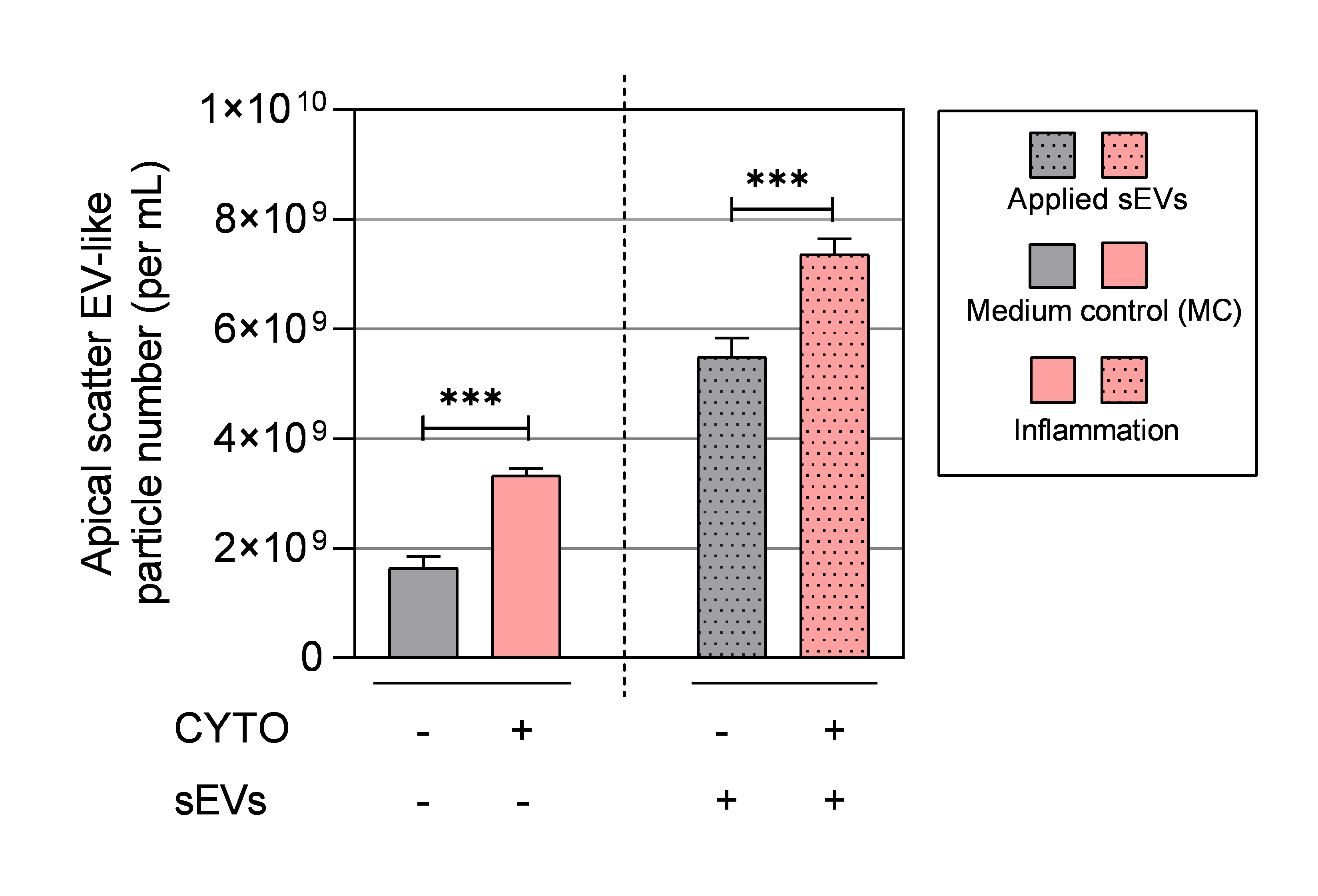


Figure S8: Inflammation induces an increase in particle release from BBB cells during incubation with PCa sEVs. After 24h incubation, apical media was collected from media with/out cytokine cocktail (CYTO) in combination with sEV (+ sEVs) derived from DU145 cells, and after a series of centrifugations steps measured with S-NTA. Where no sEVs were added (- sEVs) corresponding labelled medium control was added. (a-b) Mean ± SEM, n=15-18, N=3. Data were analysed with Grubb’s test and by one-way ANOVA followed by Holm-Šidak post-test. **p < 0.01, ***p < 0.001.
