# Supplementary material for: Uptake of DU145 and LNCaP prostate cancer cell line derived extracellular vesicles is inversely correlated with blood–brain barrier integrity in vitro
Source: Fluids Barriers CNS. 2025 Jul 7;22:70. doi: 10.1186/s12987-025-00680-7 (PMC12232752; doi:10.1186/s12987-025-00680-7)
Supplement: Supplementary file 2 — Supplementary Material 2. [file 12987_2025_680_MOESM2_ESM.docx]

**Supplementary Tables**

Table S1

List of antibodies for Western blotting.

| *Target* | *Dilution* | *Isotype* | *Conjugate* | *Clone* | *Company* | *Catalogue number* |
| --- | --- | --- | --- | --- | --- | --- |
| Alix | 1:1000 | Mouse IgG1 | unconjugated | 3A9 | Cell Signaling Technology | 2171S |
| CD81 | 1:1000 | Mouse IgG1 | unconjugated | 1G2C6 | ProteinTech GmbH | 66866-1-Ig |
| CD9 | 1:1000 | Rabbit IgG | unconjugated | D3H4P | Cell Signaling Technology | 13403S |
| TSG101 | 1:1000 | Mouse IgG1 | unconjugated | 2B7G8 | ProteinTech GmbH | 67381-1-Ig |
| GM130 | 1:1000 | Rabbit IgG | unconjugated | Not applicable | ProteinTech GmbH | 11308-1-AP |
| β-actin | 1:20000 | Mouse IgG1 | Peroxidase conjugate | AC-15 | Sigma-Aldrich | A3854 |
| Mouse IgG | 1:5000 | Horse | Peroxidase conjugate | Not applicable | Cell Signaling Technology | 7076 |
| Rabbit IgG | 1:5000 | Donkey | Peroxidase conjugate | F(ab′)2 | GE Healthcare UK Limited | LNA934V/AH |

Alix = ALG-2-interacting Protein X, GM130 = Cis-Golgi Matrix Protein 130, TSG101 = Tumour Susceptibility Gene 101.

Table S2

List of applied human primer sequences of inflammatory targets for qPCR.

| *Primer* | *Forward* | *Reverse* |
| --- | --- | --- |
| 18SrRNA | ATGGTTCCTTTGGTCGCTCG | GAGCTCACCGGGTTGGTTTT |
| β-actin/ ACTB | ACAGAGCCTCGCCTTTGC | GATATCATCATCCATGGTGAGCTG |
| ICAM1 tvb | TTCGTGTCCTGTATGGCCC | CTGGCACATTGGAGTCTGCT |
| VCAM1 tva | TGGATAATGTTTGCAGCTTCTCAA | GTAGATGTGGTCCCCTCATTCG |
| NF-κB RELA | ACTGTTCCCCCTCATCTTCC | TGGTCCTGTGTAGCCATTGA |
| JAK1 tva | TGACCGTCACCTGCTTTGAG | GGTTGGAGATTTCTCGGGGC |
| JAK1 tvb | GGGATATTTCCCTGGCCTTCT | AAGAGATCCAGAGGACCCCC |
| JAK1 tvc | CTTTGCCCTGTATGACGAGAAC | ACCTCATCCGGTAGTGGAGC |
| TRAF2 tvb | AAAGCAGTTCGGCCTTCCC | TCCTTTTCACCAAGGCGGAC |
| TRAF2 tvc | CCTTCCCAGATAATGCTGCCC | GCTCTCGTATTCTTTCAGGGTC |

18SrRNA = 18S ribosomal RNA, ICAM1 tv = Intercellular Adhesion Molecule 1 transcript variant, JAK1 = Janus Kinase 1, NF-κB = nuclear factor κB, TRAF2 = TNF Receptor Associated Factor 2, VCAM1 = Vascular Cell Adhesion Molecule 1.

Table S3

List of high-throughput qPCR barrier chip markers.

| Endogenous controls | PPIA | ACTB | GADPH | B2M |  |  |
| --- | --- | --- | --- | --- | --- | --- |
| Claudins | CLDN1 | CLDN2 | CLDN3 | CLDN4 | CLDN5 | CLDN6 |
|  | CLDN7 | CLDN8 | CLDN9 | CLDN10 tva | CLDN10 tvb | CLDN11 |
|  | CLDN12 tv1 | CLDN12 tv2 | CLDN12 tv3 | CLDN14 | CLDN15 | CLDN16 |
|  | CLDN17 | CLDN18 tv1b | CLDN18 tv2a | CLDN19 | CLDN20 | CLDN22 |
|  | CLDN23 | CLDN24 | CLDN25 | CLDN25 tv1 | CLDN25 tv7 | CLDN26 |
|  | CLDN27 |  |  |  |  |  |
| Other tight junctions | JAM-1 | JAM-2 | JAM-3 | ZO-1 | ZO-2 | ZO-3 |
|  | occludin (OCLN) | tricellulin (TRIC) |  |  |  |  |
| BBB markers | VWF | CDH1 | CDH5 | VEGFA tv4 | VEGFA tv10 | Vimentin (VIM1) |
|  | Fibronectin (FN1) | β-catenin (CTNNB1) | MFSD2A | PECAM1 |  |  |
| Transporter proteins | ABCA1 | ABCA7 | P-gp (ABCB1) | MRP1  (ABCC1) | MRP2 (ABCC2) | MRP3 (ABCC3) |
|  | MRP4 (ABCC4) | MRP5 (ABCC5) | BCRP (ABCG2) | CAT1 (SLC7A1) | CAT3 (SLC7A1) | GLUT1 (SLC2A1) |
|  | LAT1 (SLC5A7) | MCT1 (SLC16A1) | MCT8 (SLC16A2) | ENT1 (SLC29A1) |  |  |
| Receptors | TF | INSR | LRP1 | LRP8 | Angulin-1 (LSR) |  |
|  | RARA  tv1-4 | RARB tva | RARB tvb | RXRA tv1 | RXRB tv2 | RAGE tva |
| Cytokeratins | CK8 | CK18 | CK19 |  |  |  |
| Aquaporins | AQP3 | AQP5 | AQP10 | AQP11 |  |  |
| Mucins | MUC1A | MUC1B | MUC18 | MUC20 |  |  |
| Miscellaneous | S100A4 tv1 | S100A4 tv2 | APOE | WWC2 |  |  |

ABC = ATP-binding cassette, ACTB = β-actin, APOE = Apolipoprotein E, AQP = Aquaporin, B2M = β2-Microglobulin, BCRP = Breast Cancer Resistance Protein, CAT1 = catalase 1, CDH  = Cadherin, CK = Cytokeratin, CLDN = Claudin, ENT1 = Equilibrative nucleoside transporter 1, GAPDH = Glyceraldehyde-3-phosphate dehydrogenase, GLUT1 = Glucose Transporter Type 1, INSR = Insulin receptor, JAM = Junctional Adhesion Molecule, LAT1 = Linker For Activation Of T Cells 1, LRP = Low density lipoprotein (LDL) receptor-related protein, LSR = Lipolysis Stimulated Lipoprotein Receptor, MCT = Monocarboxylate-transporter, MFSD2 = Major facilitator superfamily domain-containing protein 2, MRP = Multidrug Resistance-Associated Protein, MUC = Mucin, PECAM1, Platelet And Endothelial Cell Adhesion Molecule 1, P-gp = P-glycoprotein, PPIA = Peptidylprolyl isomerase A, RAGE = Receptor for Advanced Glycation Endproducts, RAR = Retinoic Acid Receptor, RXR = Retinoid X Receptor, SLC = Solute carrier, TF = Transferrin receptor, tv = transcript variant, VEFG = Vascular endothelial growth factor, VWF = Von-Willebrand-factor, WWC2  = WW Domain-Containing Protein 2, ZO = *Zonula occludens*.

Table S4

Characterisation of sEVs was conducted using Nanoparticle Tracking Analysis (NTA). Median size (nm), particle concentration (per mL), and zeta potential (mV) of DU145, LNCaP parental sEVs derived from prostate cancer cells were measured in scatter mode. HEK293 GFP-GPI transfected sEVs were quantified in both scatter and fluorescent modes. Each sample (1 mL) diluted in distilled water was injected before sEV characterisation on Western blotting. A unique dilution for each type of sEVs was used to observe 50-200 particles per frame. Mean ± SD, 3 technical replicates of the same samples before further characterising sEVs by Western blotting.

| Small EVs | Median size (nm) | Concentration  (particles x mL^-1^) | Zeta potential (mV) |
| --- | --- | --- | --- |
| DU145 | 145.83±0.93 | 3.70E+11±8.94E+09 | -21.62±1.64 |
| LNCaP | 137.80±0.66 | 1.74E+11±6.97E+09 | -20.46±0.94 |
| HEK GFP-GPI - scatter | 138.30±2.73 | 1.56E+11±5.31E+10 | -21.09±1.77 |
| HEK GFP-GPI - fluorescent | 111.30±6.80 | 1.37E+11±5.45E+10 |  |

Table S5

Measurements of sEV-cell interaction study-related solutions: DPBS vehicle controls, essential labelled medium controls, EBM-2 basal medium, as well as isolated and CellTracker™ Orange (CTO)-stained DU145 and LNCaP sEV solutions, or HEK293 GFP-GPI transfected sEVs before their application onto cells. Particle concentration (per mL) and labelling/fluorescence efficiency (%) were measured (injected as 1-mL distilled water diluted solution) and calculated for each sample. A unique dilution for each type of sEVs allowed observation of 50-200 particles per frame. Mean ± SD, n=3-5.

| Controls for sEV-cell interactions  in distilled water/EBM-2 | Measurement mode | Concentration  (particles x mL^-1^) | | | Labelling  efficiency (%) | | |
| --- | --- | --- | --- | --- | --- | --- | --- |
| DPBS control (vehicle control) | Scatter | 0.00E+00 | ± | 0.00E+00 |  |  |  |
| DPBS control (vehicle control) | Fluorescent | 0.00E+00 | ± | 0.00E+00 | 0% | ± | 0% |
| DMEM, medium control | Scatter | 1.70E+06 | ± | 1.32E+06 |  |  |  |
| DMEM, medium control, CTO labelled | Fluorescent | 0.00E+00 | ± | 0.00E+00 | 0% | ± | 0% |
| DU145 sEV | Scatter | 8.63E+11 | ± | 2.00E+11 |  |  |  |
| DU145 sEV, CTO labelled | Fluorescent | 5.16E+11 | ± | 7.18E+10 | 60% | ± | 11% |
| RPMI medium control | Scatter | 3.50E+06 | ± | 2.41E+06 |  |  |  |
| RPMI, medium control, CTO labelled | Fluorescent | 0.00E+00 | ± | 0.00E+00 | 0% | ± | 0% |
| LNCaP sEV | Scatter | 8.03E+10 | ± | 2.75E+09 |  |  |  |
| LNCaP sEV, CTO labelled | Fluorescent | 6.33E+10 | ± | 1.53E+09 | 79% | ± | 2% |
| HEK293 GFP-GPI sEV | Scatter | 3.50E+11 | ± | 1.50E+10 |  |  |  |
| HEK293 GFP-GPI sEV, GFP+ | Fluorescent | 2.66E+11 | ± | 6.84E+10 | 76% | ± | 25% |
|  | | | | | | | |
| BBB cell culture medium | Measurement mode | Concentration  (particles x mL^-1^) | | | Labelling  efficiency (%) | | |
| EBM-2 basal medium | Scatter | 0.00E+00 | ± | 0.00E+00 |  |  |  |
| EBM-2 basal medium | Fluorescent | 0.00E+00 | ± | 0.00E+00 | 0% | ± | 0% |

Table S6

Gene expression of housekeeping and target markers from the DU145 sEV-cell interaction studies including inflammation treated samples (CYTO) and the respective medium controls (MC) using high-throughput qPCR barrier chip. List of BBB chip average values shown as mean ± SD. N=3, n=3. Data were analysed separately for each single target by Kruskal-Wallis test followed by Dunn’s multiple comparison test. (*p<0.05). Abbreviations of genes are found in Table S3.

| **Fold-change** | **DU145 sEVs** | | | **DU145 sEVs, CYTO** | | | **DU145 MC** | | | **DU145 MC, CYTO** | | |
| --- | --- | --- | --- | --- | --- | --- | --- | --- | --- | --- | --- | --- |
| **PPIA** | 1.00 | ± | 0.00 | 0.99 | ± | 0.18 | 3.02 | ± | 0.94 | 1.30 | ± | 0.24 |
| **ACTB** | 1.00 | ± | 0.00 | 0.92 | ± | 0.06 | 3.92 | ± | 1.11 | 1.55 | ± | 0.28 |
| **GAPDH** | 1.00 | ± | 0.00 | 0.97 | ± | 0.01 | 0.53 | ± | 0.19 | 0.73 | ± | 0.09 |
| **B2M** | 1.00 | ± | 0.00 | 1.13 | ± | 0.12 | 0.60 | ± | 0.10 | 0.93 | ± | 0.11 |
| **CLDN1** | 1.00 | ± | 0.00 | 0.95 | ± | 0.14 | 0.34 | ± | 0.09 | 0.85 | ± | 0.09 |
|  | Compared to MC: * | | |  | | |  | | |  | | |
| **CLDN3** | 1.00 | ± | 0.00 | 1.82 | ± | 1.04 | 1.25 | ± | 0.15 | 1.33 | ± | 0.36 |
| **CLDN4** | 1.00 | ± | 0.00 | 0.82 | ± | 0.18 | 0.77 | ± | 0.01 | 1.18 | ± | 0.18 |
| **CLDN5** | 1.00 | ± | 0.00 | 1.54 | ± | 0.38 | 0.48 | ± | 0.03 | 1.25 | ± | 0.12 |
| **CLDN6** | 1.00 | ± | 0.00 | 1.29 | ± | 0.59 | 1.26 | ± | 0.38 | 0.90 | ± | 0.35 |
| **CLDN7** | 1.00 | ± | 0.00 | 0.90 | ± | 0.14 | 0.47 | ± | 0.15 | 0.67 | ± | 0.06 |
| **CLDN11** | 1.00 | ± | 0.00 | 0.95 | ± | 0.12 | 0.55 | ± | 0.16 | 0.78 | ± | 0.19 |
| **CLDN12 tv1** | 1.00 | ± | 0.00 | 1.21 | ± | 0.26 | 0.54 | ± | 0.09 | 1.18 | ± | 0.28 |
| **CLDN12 tv2** | 1.00 | ± | 0.00 | 1.51 | ± | 0.34 | 0.64 | ± | 0.09 | 1.55 | ± | 0.40 |
| **CLDN12 tv3** | 1.00 | ± | 0.00 | 1.66 | ± | 0.29 | 0.58 | ± | 0.09 | 1.50 | ± | 0.36 |
| **CLDN14** | 1.00 | ± | 0.00 | 4.15 | ± | 1.10 | 0.91 | ± | 0.26 | 4.68 | ± | 1.65 |
| **CLDN15** | 1.00 | ± | 0.00 | 0.84 | ± | 0.27 | 1.06 | ± | 0.28 | 1.24 | ± | 0.45 |
| **CLDN16** | 1.00 | ± | 0.00 | 0.71 | ± | 0.13 | 0.69 | ± | 0.09 | 0.67 | ± | 0.09 |
| **CLDN20** | 1.00 | ± | 0.00 | 0.45 | ± | 0.33 | 0.25 | ± | 0.09 | 1.17 | ± | 0.40 |
| **CLDN22** | 1.00 | ± | 0.00 | 1.06 | ± | 0.12 | 1.14 | ± | 0.07 | 1.01 | ± | 0.02 |
| **CLDN24** | 1.00 | ± | 0.00 | 0.81 | ± | 0.24 | 1.91 | ± | 0.27 | 1.89 | ± | 0.85 |
| **F11R** | 1.00 | ± | 0.00 | 1.00 | ± | 0.02 | 0.69 | ± | 0.07 | 0.92 | ± | 0.04 |
| **JAM2** | 1.00 | ± | 0.00 | 0.57 | ± | 0.17 | 0.72 | ± | 0.27 | 0.48 | ± | 0.11 |
| **JAM3** | 1.00 | ± | 0.00 | 0.76 | ± | 0.07 | 0.45 | ± | 0.22 | 0.63 | ± | 0.12 |
| **TJP1** | 1.00 | ± | 0.00 | 0.92 | ± | 0.04 | 0.69 | ± | 0.14 | 0.95 | ± | 0.04 |
| **TJP2** | 1.00 | ± | 0.00 | 0.66 | ± | 0.04 | 0.86 | ± | 0.13 | 0.67 | ± | 0.07 |
| **VWF** | 1.00 | ± | 0.00 | 1.20 | ± | 0.60 | 0.94 | ± | 0.07 | 1.37 | ± | 0.40 |
| **SLC2A1** | 1.00 | ± | 0.00 | 1.10 | ± | 0.10 | 0.41 | ± | 0.11 | 0.81 | ± | 0.09 |
| **OCLN** | 1.00 | ± | 0.00 | 0.93 | ± | 0.09 | 0.48 | ± | 0.09 | 1.04 | ± | 0.17 |
| **CDH5** | 1.00 | ± | 0.00 | 1.17 | ± | 0.44 | 0.86 | ± | 0.11 | 1.12 | ± | 0.28 |
| **ABCB1** | 1.00 | ± | 0.00 | 0.54 | ± | 0.03 | 1.18 | ± | 0.20 | 0.69 | ± | 0.04 |
| **ABCC1** | 1.00 | ± | 0.00 | 1.01 | ± | 0.12 | 2.26 | ± | 0.66 | 1.23 | ± | 0.29 |
| **ABCC2** | 1.00 | ± | 0.00 | 1.58 | ± | 0.85 | 1.21 | ± | 0.45 | 1.86 | ± | 0.88 |
| **ABCC3** | 1.00 | ± | 0.00 | 1.39 | ± | 0.41 | 0.58 | ± | 0.15 | 1.29 | ± | 0.27 |
| **ABCC4** | 1.00 | ± | 0.00 | 0.87 | ± | 0.02 | 0.98 | ± | 0.04 | 0.93 | ± | 0.09 |
| **ABCC5** | 1.00 | ± | 0.00 | 0.84 | ± | 0.05 | 2.46 | ± | 0.71 | 1.06 | ± | 0.04 |
| **ABCG2** | 1.00 | ± | 0.00 | 0.64 | ± | 0.07 | 0.61 | ± | 0.14 | 0.57 | ± | 0.04 |
| **MARVELD2** | 1.00 | ± | 0.00 | 0.68 | ± | 0.08 | 1.67 | ± | 0.39 | 0.70 | ± | 0.08 |
| **SLC7A1** | 1.00 | ± | 0.00 | 0.88 | ± | 0.09 | 0.56 | ± | 0.10 | 0.90 | ± | 0.14 |
| **SLC29A1** | 1.00 | ± | 0.00 | 0.76 | ± | 0.10 | 0.48 | ± | 0.15 | 0.64 | ± | 0.09 |
| **INSR** | 1.00 | ± | 0.00 | 0.96 | ± | 0.09 | 0.65 | ± | 0.10 | 0.89 | ± | 0.14 |
| **SLC7A5** | 1.00 | ± | 0.00 | 0.98 | ± | 0.16 | 0.55 | ± | 0.11 | 0.94 | ± | 0.24 |
| **LRP1** | 1.00 | ± | 0.00 | 0.85 | ± | 0.35 | 0.61 | ± | 0.07 | 0.57 | ± | 0.11 |
| **LRP8** | 1.00 | ± | 0.00 | 0.89 | ± | 0.14 | 0.54 | ± | 0.08 | 0.97 | ± | 0.15 |
| **SLC16A1** | 1.00 | ± | 0.00 | 1.07 | ± | 0.17 | 0.53 | ± | 0.11 | 1.04 | ± | 0.18 |
| **SLC16A2** | 1.00 | ± | 0.00 | 1.10 | ± | 0.22 | 0.41 | ± | 0.12 | 0.88 | ± | 0.19 |
| **TF** | 1.00 | ± | 0.00 | 0.85 | ± | 0.03 | 0.33 | ± | 0.16 | 0.79 | ± | 0.13 |
| **VEGFA** | 1.00 | ± | 0.00 | 2.00 | ± | 0.39 | 0.79 | ± | 0.21 | 1.80 | ± | 0.56 |
| **LSR** | 1.00 | ± | 0.00 | 0.60 | ± | 0.20 | 0.88 | ± | 0.13 | 0.59 | ± | 0.19 |
| **WWC2** | 1.00 | ± | 0.00 | 0.95 | ± | 0.12 | 0.46 | ± | 0.09 | 0.92 | ± | 0.20 |
| **KRT8** | 1.00 | ± | 0.00 | 0.64 | ± | 0.03 | 1.50 | ± | 0.42 | 0.52 | ± | 0.11 |
| **KRT18** | 1.00 | ± | 0.00 | 0.69 | ± | 0.23 | 0.42 | ± | 0.06 | 0.47 | ± | 0.13 |
| **KRT19** | 1.00 | ± | 0.00 | 0.71 | ± | 0.30 | 3.77 | ± | 1.33 | 0.55 | ± | 0.18 |
| **AQP3** | 1.00 | ± | 0.00 | 0.60 | ± | 0.13 | 0.57 | ± | 0.07 | 0.50 | ± | 0.13 |
| **AQP11** | 1.00 | ± | 0.00 | 0.72 | ± | 0.06 | 1.17 | ± | 0.21 | 0.54 | ± | 0.09 |
| **MUC1/A** | 1.00 | ± | 0.00 | 2.09 | ± | 0.52 | 0.89 | ± | 0.10 | 1.26 | ± | 0.58 |
| **MUC1/B** | 1.00 | ± | 0.00 | 1.88 | ± | 0.32 | 3.69 | ± | 1.50 | 1.12 | ± | 0.32 |
| **MCAM** | 1.00 | ± | 0.00 | 0.86 | ± | 0.25 | 0.61 | ± | 0.07 | 0.49 | ± | 0.08 |
| **CTNNB1** | 1.00 | ± | 0.00 | 1.05 | ± | 0.14 | 1.47 | ± | 0.40 | 0.75 | ± | 0.23 |
| **VIM** | 1.00 | ± | 0.00 | 0.84 | ± | 0.07 | 1.67 | ± | 0.50 | 0.57 | ± | 0.17 |
| **FN1** | 1.00 | ± | 0.00 | 1.32 | ± | 0.12 | 0.40 | ± | 0.01 | 0.85 | ± | 0.28 |
|  |  | | | Compared to MC: * | | |  | | |  | | |
| **S100A4 tv1** | 1.00 | ± | 0.00 | 0.98 | ± | 0.55 | 1.42 | ± | 0.88 | 0.51 | ± | 0.40 |
| **CLDN25 tv1** | 1.00 | ± | 0.00 | 1.19 | ± | 0.24 | 0.88 | ± | 0.26 | 0.75 | ± | 0.22 |
| **CLDN25 tv7** | 1.00 | ± | 0.00 | 1.01 | ± | 0.35 | 0.85 | ± | 0.20 | 1.61 | ± | 0.90 |
| **APOE** | 1.00 | ± | 0.00 | 0.91 | ± | 0.19 | 0.63 | ± | 0.08 | 0.45 | ± | 0.07 |
| **RXRA tv1** | 1.00 | ± | 0.00 | 1.72 | ± | 0.51 | 4.15 | ± | 2.24 | 1.56 | ± | 0.10 |
| **RXRB tv2** | 1.00 | ± | 0.00 | 1.17 | ± | 0.19 | 0.60 | ± | 0.09 | 0.61 | ± | 0.09 |
| **MFSD2A** | 1.00 | ± | 0.00 | 2.61 | ± | 1.01 | 0.42 | ± | 0.02 | 1.76 | ± | 0.98 |
| **PECAM1** | 1.00 | ± | 0.00 | 1.16 | ± | 0.13 | 0.76 | ± | 0.15 | 0.68 | ± | 0.14 |
| **AGER** | 1.00 | ± | 0.00 | 0.89 | ± | 0.27 | 0.76 | ± | 0.15 | 0.65 | ± | 0.22 |

Table S7

Gene expression of housekeeping and target markers from the LNCaP sEV-cell interaction studies including inflammation treated samples (CYTO) and the respective medium controls (MC) using high-throughput qPCR barrier chip. List of BBB chip average values shown as mean ± SD. N=3, n=3. Data were analysed separately for each single target by Kruskal-Wallis test followed by Dunn’s multiple comparison test. (*p<0.05). Abbreviations of genes are found in Table S3.

| **Fold-change** | **LNCaP sEVs** | | | **LNCaP sEVs, CYTO** | | | **LNCaP MC** | | | **LNCaP MC, CYTO** | | |
| --- | --- | --- | --- | --- | --- | --- | --- | --- | --- | --- | --- | --- |
| **PPIA** | 1.00 | ± | 0.00 | 0.82 | ± | 0.12 | 1.26 | ± | 0.29 | 1.04 | ± | 0.28 |
| **ACTB** | 1.00 | ± | 0.00 | 1.20 | ± | 0.27 | 1.86 | ± | 0.75 | 1.44 | ± | 0.54 |
| **GAPDH** | 1.00 | ± | 0.00 | 0.82 | ± | 0.12 | 0.84 | ± | 0.17 | 0.82 | ± | 0.18 |
| **B2M** | 1.00 | ± | 0.00 | 1.25 | ± | 0.09 | 0.89 | ± | 0.12 | 1.16 | ± | 0.13 |
| **CLDN1** | 1.00 | ± | 0.00 | 0.67 | ± | 0.07 | 0.74 | ± | 0.27 | 0.63 | ± | 0.15 |
| **CLDN3** | 1.00 | ± | 0.00 | 0.61 | ± | 0.12 | 1.30 | ± | 0.46 | 0.72 | ± | 0.33 |
| **CLDN4** | 1.00 | ± | 0.00 | 0.83 | ± | 0.23 | 0.92 | ± | 0.28 | 1.16 | ± | 0.40 |
| **CLDN5** | 1.00 | ± | 0.00 | 2.57 | ± | 0.17 | 0.94 | ± | 0.35 | 2.24 | ± | 0.27 |
| **CLDN6** | 1.00 | ± | 0.00 | 2.58 | ± | 1.28 | 1.26 | ± | 0.28 | 2.36 | ± | 0.74 |
| **CLDN7** | 1.00 | ± | 0.00 | 0.62 | ± | 0.10 | 0.74 | ± | 0.22 | 0.63 | ± | 0.14 |
| **CLDN11** | 1.00 | ± | 0.00 | 0.68 | ± | 0.17 | 0.86 | ± | 0.28 | 0.60 | ± | 0.18 |
| **CLDN12 tv1** | 1.00 | ± | 0.00 | 1.60 | ± | 0.22 | 0.87 | ± | 0.23 | 1.60 | ± | 0.36 |
| **CLDN12 tv2** | 1.00 | ± | 0.00 | 1.89 | ± | 0.26 | 0.91 | ± | 0.22 | 1.76 | ± | 0.34 |
| **CLDN12 tv3** | 1.00 | ± | 0.00 | 1.94 | ± | 0.35 | 0.95 | ± | 0.33 | 1.88 | ± | 0.44 |
| **CLDN14** | 1.00 | ± | 0.00 | 9.17 | ± | 1.72 | 1.04 | ± | 0.26 | 10.06 | ± | 1.50 |
| **CLDN15** | 1.00 | ± | 0.00 | 1.30 | ± | 0.39 | 1.19 | ± | 0.59 | 1.44 | ± | 0.40 |
| **CLDN16** | 1.00 | ± | 0.00 | 0.70 | ± | 0.07 | 1.08 | ± | 0.38 | 0.71 | ± | 0.17 |
| **CLDN20** | 1.00 | ± | 0.00 | 1.21 | ± | 0.23 | 0.52 | ± | 0.16 | 0.55 | ± | 0.22 |
| **CLDN22** | 1.00 | ± | 0.00 | 1.08 | ± | 0.23 | 0.49 | ± | 0.05 | 1.21 | ± | 0.09 |
| **CLDN24** | 1.00 | ± | 0.00 | 2.75 | ± | 1.59 | 1.70 | ± | 0.32 | 1.23 | ± | 0.20 |
| **F11R** | 1.00 | ± | 0.00 | 0.97 | ± | 0.07 | 0.93 | ± | 0.16 | 0.99 | ± | 0.16 |
| **JAM2** | 1.00 | ± | 0.00 | 1.43 | ± | 0.56 | 2.16 | ± | 1.18 | 1.59 | ± | 0.79 |
| **JAM3** | 1.00 | ± | 0.00 | 0.68 | ± | 0.09 | 0.87 | ± | 0.26 | 0.68 | ± | 0.19 |
| **TJP1** | 1.00 | ± | 0.00 | 0.97 | ± | 0.13 | 0.92 | ± | 0.17 | 0.95 | ± | 0.19 |
| **TJP2** | 1.00 | ± | 0.00 | 0.74 | ± | 0.13 | 1.19 | ± | 0.29 | 0.70 | ± | 0.05 |
| **VWF** | 1.00 | ± | 0.00 | 0.93 | ± | 0.11 | 0.97 | ± | 0.03 | 0.74 | ± | 0.13 |
| **SLC2A1** | 1.00 | ± | 0.00 | 0.99 | ± | 0.16 | 0.91 | ± | 0.25 | 0.78 | ± | 0.09 |
| **OCLN** | 1.00 | ± | 0.00 | 0.88 | ± | 0.06 | 0.59 | ± | 0.13 | 0.58 | ± | 0.08 |
| **CDH5** | 1.00 | ± | 0.00 | 0.61 | ± | 0.09 | 1.19 | ± | 0.15 | 0.68 | ± | 0.05 |
|  |  | | | Compared to MC: * | | |  | | |  | | |
| **ABCB1** | 1.00 | ± | 0.00 | 0.76 | ± | 0.21 | 1.26 | ± | 0.29 | 0.75 | ± | 0.05 |
| **ABCC1** | 1.00 | ± | 0.00 | 2.07 | ± | 0.73 | 1.46 | ± | 0.26 | 2.07 | ± | 0.10 |
| **ABCC2** | 1.00 | ± | 0.00 | 1.29 | ± | 0.33 | 0.82 | ± | 0.24 | 1.35 | ± | 0.34 |
| **ABCC3** | 1.00 | ± | 0.00 | 1.32 | ± | 0.29 | 0.78 | ± | 0.23 | 1.46 | ± | 0.24 |
| **ABCC4** | 1.00 | ± | 0.00 | 1.13 | ± | 0.13 | 1.07 | ± | 0.18 | 1.14 | ± | 0.40 |
| **ABCC5** | 1.00 | ± | 0.00 | 1.08 | ± | 0.20 | 1.76 | ± | 0.39 | 1.13 | ± | 0.06 |
| **ABCG2** | 1.00 | ± | 0.00 | 0.55 | ± | 0.03 | 0.93 | ± | 0.12 | 0.51 | ± | 0.11 |
| **MARVELD2** | 1.00 | ± | 0.00 | 1.03 | ± | 0.12 | 1.46 | ± | 0.27 | 1.21 | ± | 0.03 |
| **SLC7A1** | 1.00 | ± | 0.00 | 1.04 | ± | 0.10 | 0.95 | ± | 0.18 | 0.95 | ± | 0.14 |
| **SLC29A1** | 1.00 | ± | 0.00 | 0.74 | ± | 0.06 | 0.92 | ± | 0.23 | 0.68 | ± | 0.09 |
| **INSR** | 1.00 | ± | 0.00 | 1.20 | ± | 0.13 | 1.00 | ± | 0.24 | 1.08 | ± | 0.11 |
| **SLC7A5** | 1.00 | ± | 0.00 | 1.21 | ± | 0.24 | 0.84 | ± | 0.24 | 1.51 | ± | 0.12 |
| **LRP1** | 1.00 | ± | 0.00 | 0.72 | ± | 0.20 | 1.19 | ± | 0.38 | 0.70 | ± | 0.45 |
| **LRP8** | 1.00 | ± | 0.00 | 0.89 | ± | 0.15 | 1.09 | ± | 0.44 | 1.08 | ± | 0.22 |
| **SLC16A1** | 1.00 | ± | 0.00 | 1.59 | ± | 0.13 | 0.92 | ± | 0.15 | 1.45 | ± | 0.31 |
| **SLC16A2** | 1.00 | ± | 0.00 | 1.61 | ± | 0.13 | 0.94 | ± | 0.35 | 1.25 | ± | 0.14 |
| **TF** | 1.00 | ± | 0.00 | 0.83 | ± | 0.09 | 0.81 | ± | 0.22 | 0.74 | ± | 0.27 |
| **VEGFA** | 1.00 | ± | 0.00 | 4.87 | ± | 0.82 | 1.09 | ± | 0.37 | 4.13 | ± | 0.17 |
| **LSR** | 1.00 | ± | 0.00 | 0.68 | ± | 0.13 | 1.59 | ± | 0.35 | 0.75 | ± | 0.45 |
| **WWC2** | 1.00 | ± | 0.00 | 1.16 | ± | 0.21 | 1.00 | ± | 0.33 | 1.17 | ± | 0.03 |
| **KRT8** | 1.00 | ± | 0.00 | 0.58 | ± | 0.06 | 0.82 | ± | 0.31 | 0.61 | ± | 0.31 |
| **KRT18** | 1.00 | ± | 0.00 | 0.26 | ± | 0.05 | 0.64 | ± | 0.26 | 0.27 | ± | 0.11 |
| **KRT19** | 1.00 | ± | 0.00 | 0.48 | ± | 0.06 | 1.12 | ± | 0.18 | 0.35 | ± | 0.07 |
| **AQP3** | 1.00 | ± | 0.00 | 0.43 | ± | 0.06 | 0.84 | ± | 0.29 | 0.46 | ± | 0.08 |
| **AQP11** | 1.00 | ± | 0.00 | 0.73 | ± | 0.08 | 0.83 | ± | 0.27 | 0.64 | ± | 0.11 |
| **MUC1/A** | 1.00 | ± | 0.00 | 1.06 | ± | 0.14 | 1.19 | ± | 0.52 | 1.47 | ± | 0.12 |
| **MUC1/B** | 1.00 | ± | 0.00 | 1.29 | ± | 0.18 | 1.04 | ± | 0.09 | 1.44 | ± | 0.73 |
| **MCAM** | 1.00 | ± | 0.00 | 0.60 | ± | 0.09 | 1.03 | ± | 0.43 | 0.62 | ± | 0.12 |
| **CTNNB1** | 1.00 | ± | 0.00 | 0.91 | ± | 0.08 | 0.96 | ± | 0.24 | 0.87 | ± | 0.18 |
| **VIM** | 1.00 | ± | 0.00 | 0.76 | ± | 0.14 | 0.83 | ± | 0.34 | 0.67 | ± | 0.10 |
| **FN1** | 1.00 | ± | 0.00 | 1.82 | ± | 0.50 | 1.28 | ± | 0.54 | 2.64 | ± | 0.05 |
| **S100A4 tv1** | 1.00 | ± | 0.00 | 0.86 | ± | 0.47 | 0.73 | ± | 0.23 | 0.38 | ± | 1.19 |
| **CLDN25 tv1** | 1.00 | ± | 0.00 | 1.13 | ± | 0.07 | 0.73 | ± | 0.23 | 1.17 | ± | 0.08 |
| **CLDN25 tv7** | 1.00 | ± | 0.00 | 1.56 | ± | 0.35 | 1.45 | ± | 0.55 | 1.40 | ± | 0.45 |
| **APOE** | 1.00 | ± | 0.00 | 0.58 | ± | 0.13 | 1.21 | ± | 0.43 | 0.94 | ± | 0.52 |
| **RXRA tv1** | 1.00 | ± | 0.00 | 0.64 | ± | 0.07 | 0.90 | ± | 0.44 | 1.24 | ± | 0.54 |
| **RXRB tv2** | 1.00 | ± | 0.00 | 1.04 | ± | 0.08 | 0.89 | ± | 0.29 | 1.07 | ± | 0.66 |
| **MFSD2A** | 1.00 | ± | 0.00 | 3.58 | ± | 0.73 | 0.88 | ± | 0.27 | 4.01 | ± | 0.38 |
| **PECAM1** | 1.00 | ± | 0.00 | 0.99 | ± | 0.09 | 1.06 | ± | 0.42 | 1.12 | ± | 1.53 |
| **AGER** | 1.00 | ± | 0.00 | 0.91 | ± | 0.05 | 1.23 | ± | 0.40 | 1.23 | ± | 0.44 |
